# Mechanochemical control of epidermal stem cell divisions by B-plexins

**DOI:** 10.1038/s41467-021-21513-9

**Published:** 2021-02-26

**Authors:** Chen Jiang, Ahsan Javed, Laura Kaiser, Michele M. Nava, Rui Xu, Dominique T. Brandt, Dandan Zhao, Benjamin Mayer, Javier Fernández-Baldovinos, Luping Zhou, Carsten Höß, Kovilen Sawmynaden, Arkadiusz Oleksy, David Matthews, Lee S. Weinstein, Heidi Hahn, Hermann-Josef Gröne, Peter L. Graumann, Carien M. Niessen, Stefan Offermanns, Sara A. Wickström, Thomas Worzfeld

**Affiliations:** 1 Institute of Pharmacology, University of Marburg, Marburg, Germany; 2 Department of Pharmacology, Max-Planck-Institute for Heart and Lung Research, Bad Nauheim, Germany; 3 Helsinki Institute of Life Science, Biomedicum Helsinki, University of Helsinki, Helsinki, Finland; 4 Biochemistry, Faculty of Chemistry, and Center for Synthetic Microbiology (synmikro), University of Marburg, Marburg, Germany; 5 Department of Diabetes and Endocrinology, Affiliated Hospital of Southwest Medical University, Luzhou, China; 6 LifeArc, Stevenage, UK; 7 Metabolic Diseases Branch, NIDDK, NIH, Bethesda, USA; 8 Institute of Human Genetics, University Medical Center Göttingen, Göttingen, Germany; 9 Cologne Excellence Cluster on Cellular Stress Responses in Aging-Associated Diseases (CECAD), Center for Molecular Medicine Cologne, University of Cologne, Cologne, Germany; 10 Medical Faculty, University of Frankfurt, Frankfurt, Germany; 11 Wihuri Research Institute, Biomedicum Helsinki, University of Helsinki, Helsinki, Finland; 12 Stem Cells and Metabolism Research Program, Faculty of Medicine, University of Helsinki, Helsinki, Finland; 13 Max-Planck-Institute for Biology of Ageing, Cologne, Germany

**Keywords:** Basal cell carcinoma, Mitosis, Cell proliferation, Skin stem cells

## Abstract

The precise spatiotemporal control of cell proliferation is key to the morphogenesis of epithelial tissues. Epithelial cell divisions lead to tissue crowding and local changes in force distribution, which in turn suppress the rate of cell divisions. However, the molecular mechanisms underlying this mechanical feedback are largely unclear. Here, we identify a critical requirement of B-plexin transmembrane receptors in the response to crowding-induced mechanical forces during embryonic skin development. Epidermal stem cells lacking B-plexins fail to sense mechanical compression, resulting in disinhibition of the transcriptional coactivator YAP, hyperproliferation, and tissue overgrowth. Mechanistically, we show that B-plexins mediate mechanoresponses to crowding through stabilization of adhesive cell junctions and lowering of cortical stiffness. Finally, we provide evidence that the B-plexin-dependent mechanochemical feedback is also pathophysiologically relevant to limit tumor growth in basal cell carcinoma, the most common type of skin cancer. Our data define a central role of B-plexins in mechanosensation to couple cell density and cell division in development and disease.

## Abstract (web summary)

It is unclear how epithelial tissues adjust cell division rates to cell density. Here, the authors show that Plexin-B1 and Plexin-B2 sense mechanical compression (crowding) of epidermal stem cells, resulting in inactivation of YAP and suppression of cell proliferation.

## Introduction

During embryogenesis, tissue growth and shape are intimately connected^1^. Tissue growth intrinsically generates mechanical strains and compressions, and, conversely, mechanical deformations provide a fundamental regulatory feedback for growth control^1–3^. This feedback requires cells to sense mechanical forces, which are then transformed into biochemical signals that in turn control cellular functions including the rate of cell divisions^3–6^. Compression of cells within a tissue results in suppression of proliferation, a response termed contact inhibition of proliferation^2,7^. In the mammalian embryonic epidermis, proliferation of stem cells within the mechanically jammed basal layer causes crowding and local cell stress anisotropy^8–10^. How epidermal stem cells sense these mechanical forces and couple them with division is largely unknown.

Plexins constitute a family of single-pass transmembrane proteins that have initially been described for their role as axon guidance receptors for semaphorins in the developing nervous system^11,12^. In vertebrates, nine plexins have been identified, which—based on homology—are grouped into four subfamilies, Plexin-A1–4, Plexin-B1–3, Plexin-C1, and Plexin-D1^13^. They are now recognized to be of central importance for cell–cell communication in multiple tissues and biological systems, such as the immune and bone system, as well as in cancer^14–16^. Recently, Plexin-D1 has been shown to be activated by fluid shear stress-induced mechanical forces in vascular endothelial cells^17^. Whether plexins play a role in mechanosensation in epithelial cells is unknown.

Here, we report that epidermal stem cells require Plexin-B1 and Plexin-B2 to sense mechanical compression, and uncover Plexin-B1 and Plexin-B2 as negative upstream regulators of YAP that suppress stem cell divisions in response to crowding. This biomechanical feedback mechanism ensures a robust control of proliferation rates by cell density, and is functionally relevant for both tissue morphogenesis as well as cancer.

## Results

### Plexin-B1/Plexin-B2 suppress epidermal stem cell proliferation during embryonic development

**Fig. 1:**
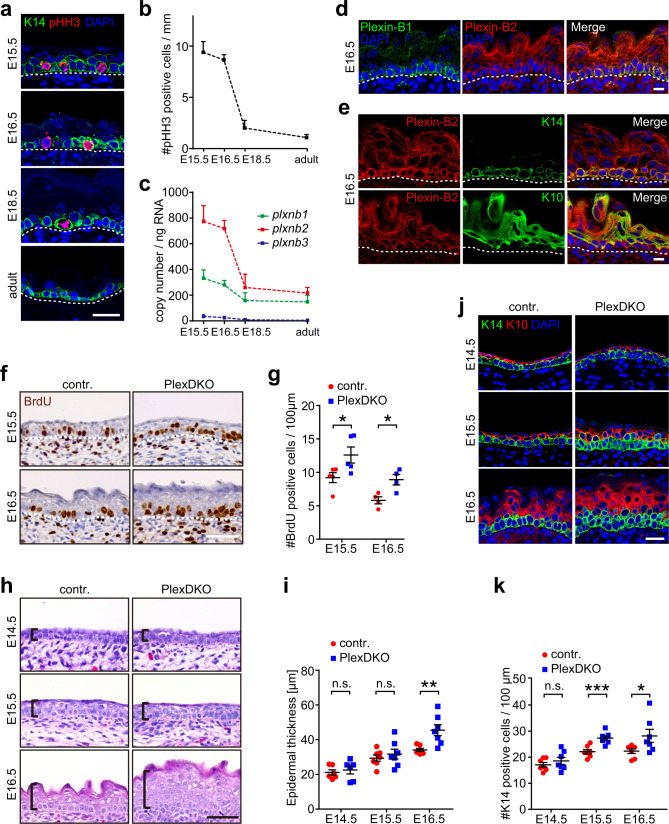
Plexin-B1/Plexin-B2 suppress epidermal stem cell proliferation during embryonic development. **a** Confocal images of immunostainings of murine skin at embryonic day 15.5 (E15.5), 16.5 (E16.5), 18.5 (E18.5) and at an adult stage using anti-phospho-histone H3 (pHH3; red) and anti-keratin 14 (K14; green) antibodies. Dashed lines indicate the basement membrane. Scale bar, 25 µm. **b** Quantification of pHH3-positive cells at different time points of embryonic (E) development and in the adult (mean ± s.e.m.; *n* = 3 mice per time point). **c** mRNA expression levels of genes encoding B-plexins (*plxnb*) in the murine skin at the indicated time points as determined by quantitative RT-PCR (mean ± s.e.m.; *n* = 3 mice per time point). **d** Confocal images of immunostainings of murine epidermis at embryonic day 16.5 (E16.5) using anti-Plexin-B1 (green) and anti-Plexin-B2 antibodies (red). Dashed lines indicate the basement membrane. Scale bar, 10 µm. **e** Confocal images of immunostainings of murine epidermis at E16.5 using anti-Plexin-B2 (red), and anti-keratin 14 (K14) or anti-keratin 10 (K10) antibodies (green). Scale bar, 10 µm. **f** Immunohistochemistry on murine embryonic epidermis using an anti-BrdU antibody (brown). “contr.”: control mice (genotype *plxnb1*^flox/flox^;*plxnb2*^flox/flox^), “PlexDKO”: epidermis-specific Plexin-B1/Plexin-B2 double-knockout mice (genotype K14-Cre;*plxnb1*^flox/flox^;*plxnb2*^flox/flox^). Blue: Hematoxylin. Scale bar, 50 µm. **g** Quantification of the data in **f** (mean ± s.e.m.; E15.5: *n* = 5 mice per genotype, E16.5: *n* = 4 mice per genotype; *p* = 0.0429 for E15.5, *p* = 0.0175 for E16.5; two-sided unpaired *t*-test). **h** H&E-stained histological sections of murine epidermis at the indicated time points of embryonic development. Brackets indicate epidermal thickness. Scale bar, 50 µm. **i** Quantification of the data in **h** (mean ± s.e.m.; E14.5: *n* = 6 mice per genotype, E15.5 and E16.5: *n* = 7 mice per genotype and time point; *p* = 0.625 for E14.5, *p* = 0.494 for E15.5, *p* = 0.005 for E16.5; two-sided unpaired *t*-test). **j** Confocal images of immunostainings of murine embryonic epidermis using anti-K14 (green) and anti-K10 (red) antibodies. Scale bar, 25 µm. **k** Quantification of K14-positive cells (mean ± s.e.m.; *n* = 7 mice per genotype and time point; *p* = 0.38 for E14.5, *p* = 0.0009 for E15.5, *p* = 0.0497 for E16.5; two-sided unpaired *t*-test).

**Fig. 2:**
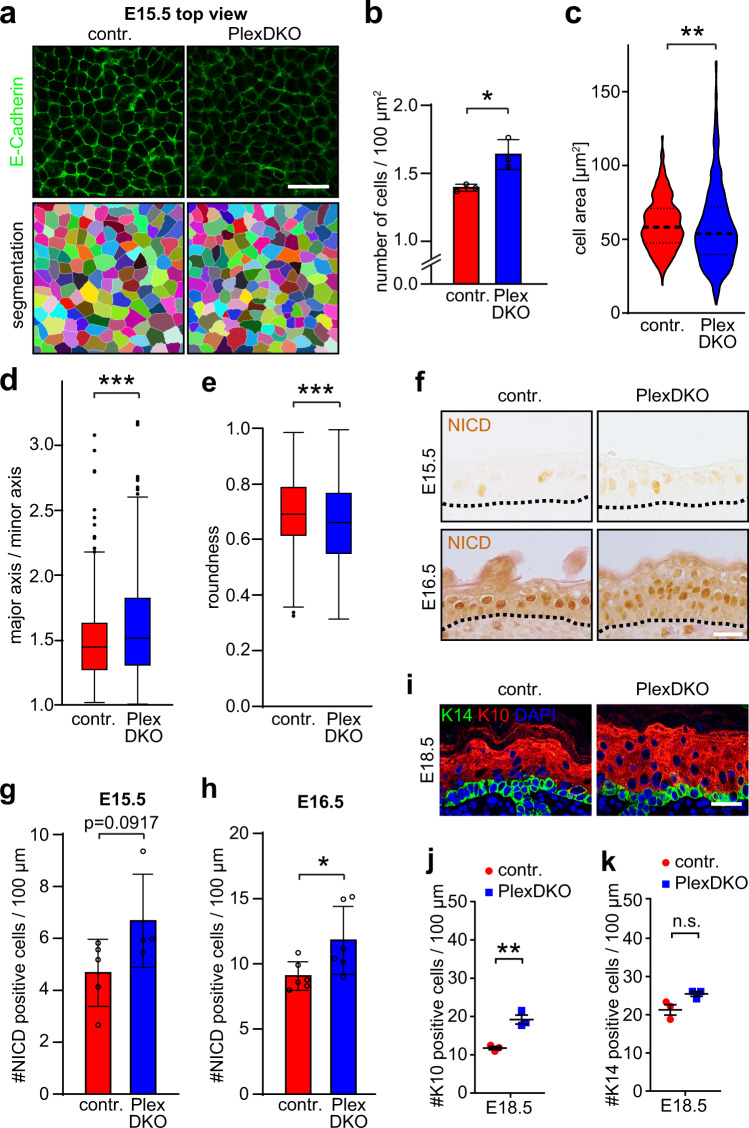
Hyperproliferation of Plexin-B1/Plexin-B2 double-deficient epidermal stem cells induces overcrowding, cell shape anisotropy and differentiation. **a** Upper row: Confocal images of whole-mount immunostainings (top view) of murine epidermis at E15.5 using an anti-E-cadherin antibody (green). Shown are representative images of the basal cell layer. Lower row: Segmentation analyses of the images depicted in the upper row. Scale bar, 25 µm. **b** Quantification of the number of epidermal stem cells (basal layer) per area (mean ± s.d.; *n* = 3 mice per genotype; *p* = 0.0199; two-sided unpaired *t*-test). **c** Analysis of epidermal stem cell areas (violin plot with first quartile, median, and third quartile; control: *n* = 415 from three mice, PlexDKO: *n* = 509 from three mice; *F*-test of equality of variances: *p* < 0.0001, two-sided Mann–Whitney *U* test: *p* = 0.0011). **d**, **e** Quantification of cell shape anisotropy (box plot with minimum, first quartile, median, third quartile and maximum; control: *n* = 415 from three mice, PlexDKO: *n* = 509 from three mice; two-sided Mann–Whitney *U* test: *p* = 0.0003). **f** Immunohistochemistry on murine embryonic epidermis using an anti-NICD antibody (brown). Dashed lines indicate the basement membrane. Scale bar, 25 µm. **g**, **h** Quantification of the data in **f** (mean ± s.d.; E15.5 control: *n* = 5 mice, E15.5 PlexDKO: *n* = 4 mice, E16.5: *n* = 6 mice per genotype; *p* = 0.0917 at E15.5, *p* = 0.0391 for E16.5; two-sided unpaired *t*-test). **i** Confocal images of immunostainings of murine epidermis at E18.5 using anti-K14 (green) and anti-K10 (red) antibodies. Scale bar, 25 µm. **j**, **k** Quantification of the data in **i** (mean ± s.e.m.; *n* = 3 mice per genotype; **j** *p* = 0.0041, **k** *p* = 0.0569, two-sided unpaired *t*-test).

Divisions of stem cells within an epithelial layer induce crowding and cell shape anisotropy^18^. We hypothesized that a mechanical feedback to control stem cell divisions would therefore be particularly relevant during developmental stages with high stem cell proliferation rates. We observed that in the developing mouse epidermis, stem cell proliferation rates were highest at embryonic day 15.5 (E15.5) and gradually declined to very low rates in the adult (Fig. 1a, b), consistent with previous reports^19,20^. The mRNA expression levels of plexins showed a striking positive correlation with proliferation rates, with Plexin-B2 being the most highly expressed plexin family member during embryonic development (Fig. 1c and Supplementary Fig. 1a, b). Immunostainings revealed that Plexin-B2 was expressed in all layers of the developing epidermis including the K14-positive stem cells (Fig. 1d, e). Plexin-B1, a receptor highly homologous to Plexin-B2^11,13^, localized exclusively to the stem cell layer (Fig. 1d). To identify the functional role of Plexin-B1 and Plexin-B2 in epidermal stem cell proliferation, we inactivated the respective genes. Given (1) the overlapping expression pattern of Plexin-B1 and Plexin-B2 in K14-positive stem cells of the epidermis (Fig. 1d), (2) the functional redundancy of these two receptors during development of other tissues^21–23^, and (3) our observation that Plexin-B1 single-deficient and Plexin-B2 single-deficient embryos were devoid of any epidermal abnormalities (Supplementary Fig. 1c–f), we generated mice lacking both Plexin-B1 and Plexin-B2 in epidermal stem cells by crossing conditional alleles for Plexin-B1 and Plexin-B2 with a constitutively expressed keratin 14-Cre line (K14-Cre;*plxnb1*^flox/flox^;*plxnb2*^flox/flox^). Consistent with the onset of Cre expression^24^, Plexin-B1 and Plexin-B2 expression was lost as early as E15.5 (Supplementary Fig. 1g). Interestingly, Plexin-B1/Plexin-B2 double-knockout mice (“PlexDKO”) showed a marked epidermal hyperproliferation (Fig. 1f, g), resulting in epidermal thickening (Fig. 1h, i). This epidermal thickening was due to an expansion of K14-positive stem cells, while the number of K10-positive differentiated cells was not significantly increased (Fig. 1j, k and Supplementary 1h). Apoptosis rates remained unchanged (Supplementary Fig. 1i). Taken together, these results indicate that Plexin-B1 and Plexin-B2 suppress epidermal stem cell proliferation during embryonic development.

### Hyperproliferation of Plexin-B1/Plexin-B2 double-deficient epidermal stem cells induces overcrowding, cell shape anisotropy and differentiation

We next investigated how the increased number of epidermal stem cell divisions in Plexin-B1/Plexin-B2 double-knockout mice affected stem cell density and shape. To do so, we performed segmentation analyses of the epidermal stem cell layer at embryonic day 15.5 (Fig. 2a). In mice lacking Plexin-B1 and Plexin-B2, we observed both a higher cell density (Fig. 2b) as well as smaller and more variable cell sizes (Fig. 2c) than in control mice. This was accompanied by an increase in cell shape anisotropy, with cells being more elongated (Fig. 2d, e). Similar abnormalities were detected at embryonic day 16.5 (Supplementary Fig. 2a–e). These data demonstrate that Plexin-B1/Plexin-B2 double-deficiency results in overcrowding and shape deformation in the epidermal stem cell layer.

It is known that proliferation-induced crowding and cell shape distortion trigger differentiation and delamination of epidermal stem cells^18^. To assess whether loss of Plexin-B1/Plexin-B2 affects stem cell differentiation, we performed immunostainings for active Notch (Notch intracellular domain (NICD)), which plays a decisive role in epidermal differentiation and specifically labels epidermal cells committed to differentiation^25^. While the number of NICD-positive cells in the Plexin-B1/Plexin-B2-deficient epidermis was not significantly changed at embryonic day 15.5 (Fig. 2f, g and Supplementary Fig. 2f)—the developmental stage when stem cell hyperproliferation was most pronounced (Fig. 1f, g)—Notch signaling became markedly increased at embryonic day 16.5 (Fig. 2f, h). This increase was due to elevated numbers of NICD-positive cells in suprabasal layers of the epidermis (Supplementary Fig. 2g). Consistently, we found an expansion of K10-positive differentiating cells in suprabasal layers at later embryonic stages, i.e. at E18.5 (Fig. 2i, j and Supplementary Fig. 2h, i). Concomitantly, the number of K14-positive stem cells in the Plexin-B1/Plexin-B2-deficient epidermis normalized at E18.5 (Fig. 2i, k). As no differences in apoptosis rate were detected (Supplementary Fig. 2j), we conclude that hyperproliferation of Plexin-B1/Plexin-B2-deficient epidermal stem cells coincides with overcrowding and cell shape anisotropy in the basal layer, which is followed—with a temporal delay—by increased differentiation to restore tissue size and architecture.

### Plexin-B1/Plexin-B2 inhibit YAP activity in response to mechanical forces

**Fig. 3:**
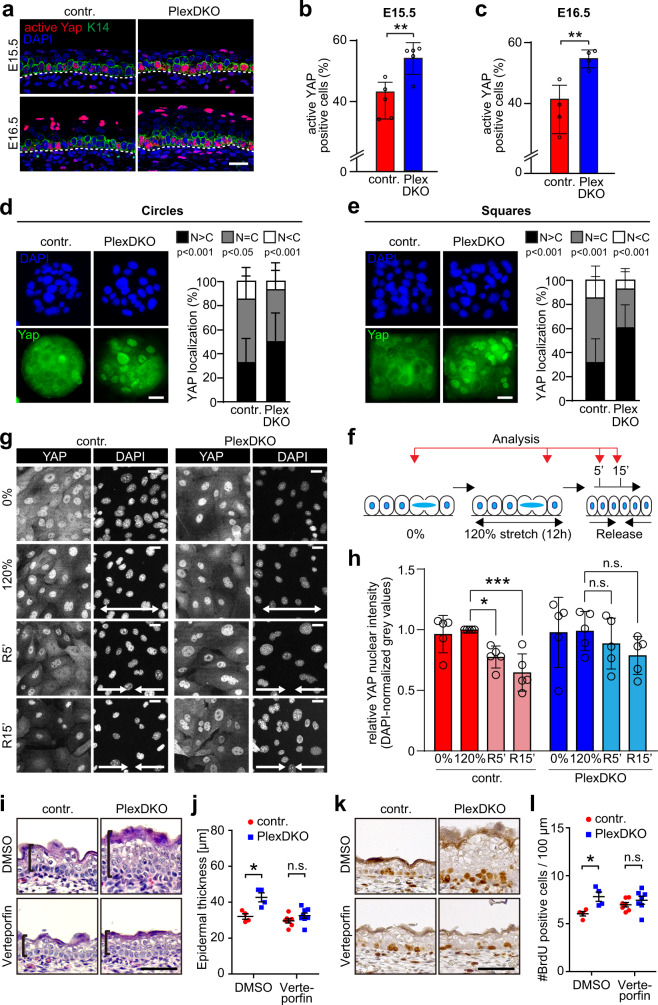
Plexin-B1/Plexin-B2 inhibit YAP activity in response to mechanical forces. **a** Confocal images of murine epidermis at E15.5 and E16.5 immunostained for active YAP (red) and K14 (green). Scale bar, 25 µm. **b**, **c** Quantification of the data in **a** (percentage of basal cells) (mean ± s.d.; E15.5: *n* = 5 mice per genotype, E16.5: *n* = 4 mice per genotype; **b** *p* = 0.0047, **c** *p* = 0.0078; two-sided unpaired *t*-test). **d**, **e** Primary mouse keratinocytes on **d** circular or **e** square micropatterns immunostained for Yap (green) after 3 h of 1.8 mM Ca^2+^. Representative images are shown on the left, quantifications of Yap localization are shown on the right (mean ± s.d.; circles: control: *n* = 73 micropatterns from 5 mice, PlexDKO: *n* = 73 micropatterns from four mice; N > C: *p* = 0.000008, N = C: *p* = 0.012038, N < C: *p* = 0.000008; squares: control: *n* = 69 micropatterns from five mice, PlexDKO: *n* = 69 micropatterns from four mice; N > C: *p* < 0.000001, N = C: *p* < 0.000001, N < C: *p* = 0.000050; two-sided unpaired *t*-test). Blue: DAPI. Scale bars, 25 μm. **f** Schematic illustration of abrupt crowding experiments. Confluent monolayers were exposed to 120% static uniaxial stretch, which was abruptly released. Cells were analyzed at the indicated time points (R5’: 5 min after release; R15’: 15 min after release). **g**, **h** Representative YAP immunofluorescence images **g** and quantification of nuclear YAP **h** upon crowding (mean ± s.d.; *n* = 5 independent experiments with >200 cells/condition/experiment; *p = 0.0358, ***p = 0.0004, one-way ANOVA, Tukey test; values are normalized to control 120% stretch; statistics have been done from the non-normalized values; scale bars 20 µm). **i**–**l** Pregnant females were injected at E15.5 with vehicle control (DMSO) or verteporfin (i.p., 100 μg/g body weight). Embryos were harvested at E16.5. Shown are in **i** representative H&E-stained histological sections, in **j** the quantification of epidermal thickness (mean ± s.e.m.; control DMSO-treated: *n* = 4 embryos, PlexDKO DMSO-treated: *n* = 4 embryos, control verteporfin-treated: *n* = 7 embryos, PlexDKO verteporfin-treated: *n* = 8 embryos; *p* = 0.011 for DMSO, *p* = 0.165 for verteporfin; two-sided unpaired *t*-test), in **k** representative images of immunohistochemical stainings of the embryonic epidermis using an anti-BrdU antibody (brown), and in **l** the quantification of BrdU-positive cells (mean ± s.e.m.; control DMSO-treated: *n* = 4 embryos, PlexDKO DMSO-treated: *n* = 4 embryos, control verteporfin-treated: *n* = 7 embryos, PlexDKO verteporfin-treated: *n* = 7 embryos; *p* = 0.016 for DMSO, *p* = 0.345 for verteporfin; two-sided unpaired *t*-test).

We next sought to identify the downstream signaling mechanism by which Plexin-B1/Plexin-B2 balance cell division rates to cell density. The transcriptional regulator YAP is known as a key factor in sensing cell density and shape to regulate cell proliferation^7,26^, and has been shown to play critical functional roles in skin development, homeostasis, expansion, repair, and cancer^27–30^. In the epidermis, high cell densities suppress YAP activity and stem cell divisions^30,31^. We therefore examined how stem cell overcrowding and shape anisotropy in the Plexin-B1/Plexin-B2-deficient embryonic epidermis impact on YAP signaling. Surprisingly, we observed that both the levels of active, dephosphorylated YAP (Fig. 3a–c) as well as the mRNA expression level of the YAP target gene *ctgf* (Supplementary Fig. 3a–c) were elevated in embryonic epidermal stem cells lacking Plexin-B1 and Plexin-B2. This suggested that Plexin-B1/Plexin-B2-deficient epidermal stem cells fail to restrict YAP activity in response to crowding. To test this hypothesis, we cultured primary mouse keratinocytes at different densities and analyzed YAP localization as well as mRNA expression levels of YAP target genes. At low cell densities, when YAP is predominantly localized to the nucleus and active, these parameters were uninfluenced by the loss of Plexin-B1/Plexin-B2 (Supplementary Fig. 3d, e). At high cell densities, however, Plexin-B1/Plexin-B2-deficiency impeded shuttling of YAP out of the nucleus (Supplementary Fig. 3h) and resulted in elevated mRNA expression levels of YAP target genes (Supplementary Fig. 3i). Interestingly, the suppressive effect of Plexin-B1/Plexin-B2 on YAP activity at high cell density was dependent on the presence of calcium in the culture medium (Supplementary Fig. 3f–i), which induces the formation of cadherin-based cell–cell adhesion complexes^32^. To further assess the role of Plexin-B1/Plexin-B2 in mechanosensation of tissue stress anisotropy caused by crowding, we seeded primary mouse keratinocytes on circular or square micropatterned surfaces with identical surface areas, on which cells experience isotropic (circles) and anisotropic (squares) traction stresses^18^. Fully in line with a function of Plexin-B1/Plexin-B2 in mechanosensation of crowding, the dependence of YAP localization on Plexin-B1/Plexin-B2 was more pronounced under anisotropic than under isotropic traction stress conditions (Fig. 3d, e). Finally, to directly test for a requirement of Plexin-B1/Plexin-B2 in mechanosensation of crowding-induced mechanical forces, we subjected primary mouse keratinocyte monolayers to static stretch (for 12 h) to expand the cell–substrate adhesive surface area, followed by release of tension resulting in a transient increase in monolayer compression^18^ (Fig. 3f). While in control cells compression triggered a reduction in nuclear YAP, Plexin-B1/Plexin-B2-deficient cells failed to respond (Fig. 3g, h).

To test whether the loss of B-plexin-mediated suppression of YAP activity in response to mechanical forces was causative for tissue overgrowth in vivo, we pharmacologically inhibited YAP in embryos. To do so, the YAP inhibitor verteporfin^33,34^ was injected into pregnant female mice at E15.5, and embryonic epidermal thickness and proliferation were analyzed 24 h later at E16.5. Indeed, verteporfin treatment largely normalized both epidermal thickness (Fig. 3i, j) and epidermal stem cell proliferation rates (Fig. 3k, l) of Plexin-B1/Plexin-B2-deficient embryos as compared to control embryos. Taken together, these data indicate that Plexin-B1/Plexin-B2 are required for mechanosensation in epidermal stem cells, and suppress YAP activity in response to mechanical forces generated by crowding-induced lateral compression. Moreover, they demonstrate causality of the B-plexin-mediated suppression of YAP activity for the limitation of epidermal tissue growth.

### Plexin-B1/Plexin-B2 mediate mechanosensation through stabilization of adhesive cell–cell junctions

**Fig. 4:**
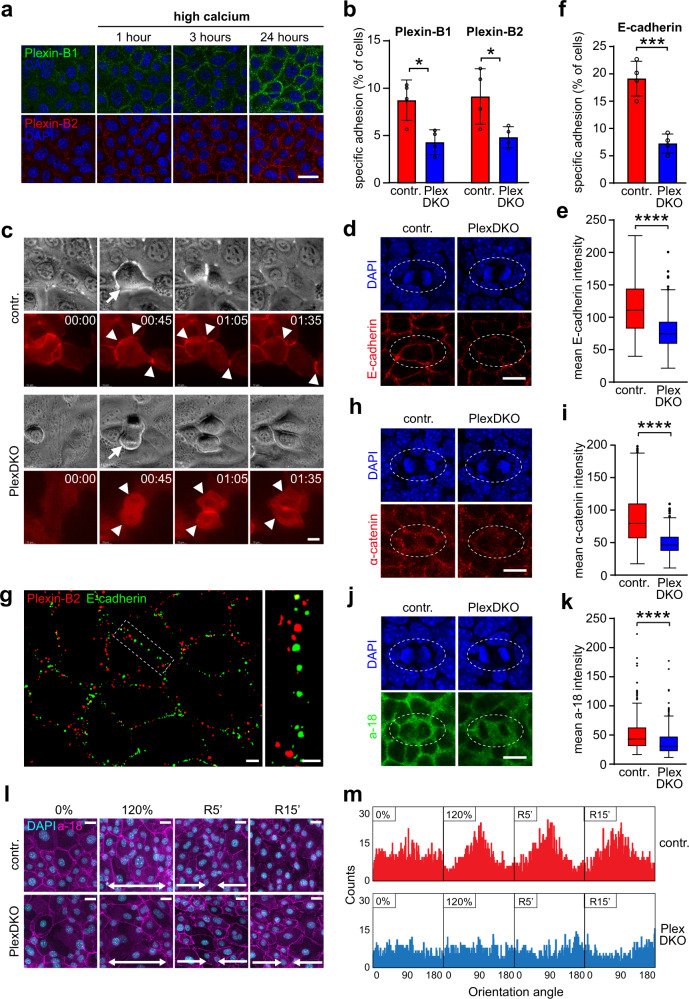
Plexin-B1/Plexin-B2 mediate mechanosensation through stabilization of adhesive cell–cell junctions. **a** Primary mouse keratinocytes were cultured with 70 µM Ca^2+^. 1.8 mM Ca^2+^ was added (“high calcium”). Shown are confocal images of immunostainings for Plexin-B1 (green) and Plexin-B2 (red). Scale bar, 25 µm. **b**, **f** Specific adhesion of primary mouse keratinocytes to the recombinant extracellular portions of **b** Plexin-B1 or Plexin-B2, and **f** E-cadherin (mean ± s.d.; *n* = 4 mice per genotype; *p* = 0.0124 for Plexin-B1, *p* = 0.0332 for Plexin-B2; *p* = 0.0006 for E-cadherin; two-sided unpaired *t*-test). **c** Live cell imaging of primary mouse keratinocytes expressing E-cadherin-mRuby. Shown are representative epifluorescence still images of cell divisions. Arrows: dividing cells; arrowheads: cell–cell contacts. Time: hours:minutes format. Scale bar, 10 µm. **d**, **h**, **j** Confocal images of the epidermal basal layer at E15.5 immunostained for **d** E-cadherin (red), **h** α-catenin (red), and **j** a-18 (=α-catenin tension-sensitive epitope antibody; green). Cell divisions are contoured by dashed lines. Scale bar, 10 µm. **e**, **i**, **k** Quantification of **e** E-cadherin, **i** α-catenin, and **k** a-18 immunofluorescence intensities at cell–cell contacts between a dividing cell and its immediate neighbors (box plot with minimum, first quartile, median, third quartile and maximum). **e** control: *n* = 207 cell–cell contacts of 34 dividing cells from three mice, PlexDKO: *n* = 209 cell–cell contacts of 34 dividing cells from three mice. **i** control: *n* = 228 cell–cell contacts of 37 dividing cells from three mice, PlexDKO: *n* = 244 cell–cell contacts of 39 dividing cells from three mice. **k** control: *n* = 222 cell–cell contacts of 36 dividing cells from three mice, PlexDKO: *n* = 219 cell-cell contacts of 37 dividing cells from three mice. Two-sided Mann–Whitney *U* test for **e**, **i**, and **k**: *p* < 0.0001. **g** Super-resolution structured illumination microscopy images of the epidermal basal layer at E15.5 immunostained for Plexin-B2 (red) and E-cadherin (green). Scale bar, 2 µm. The dashed boxed area is magnified on the right. Scale bar, 1 µm. **l** Representative a-18 (magenta) and nuclear (DAPI; cyan) immunofluorescence confocal images of primary mouse keratinocyte monolayers exposed to uniaxial static stretch and subsequent release. Scale bars, 20 µm. **m** Quantification of **l** showing the orientation angle of cell major axes perpendicular to the stretch direction (frequency distribution of >500 cells/condition pooled across three independent experiments; *K*^2^ = 366.4 (0% contr.), 5.804 (120% contr.) 164.2 (0% PlexDKO), 215.2 (120% PlexDKO); D’Agostino–Pearson Omnibus test).

We next aimed to elucidate the precise molecular mechanism through which Plexin-B1/Plexin-B2 limit mechanosensitive YAP activity and epidermal stem cell proliferation. Plexin-B1 and Plexin-B2 are known to serve as receptors for class-4 transmembrane semaphorins, and to mediate forward signaling in response to ligand binding^13^. In addition, both Plexin-B1 as well as Plexin-B2 can act as ligands and induce reverse signaling via binding to class-4 transmembrane semaphorins^35,36^. First, we examined expression levels of Plexin-B1/Plexin-B2 ligands, i.e. of semaphorin 4A (Sema4A), semaphorin 4C (Sema4C), and semaphorin 4D (Sema4D), in primary mouse keratinocytes and found these to be largely unaltered by Plexin-B1/Plexin-B2 deficiency (Supplementary Fig. 4a–c). To investigate the effect of an activation of semaphorin-plexin signaling on YAP activity, we treated primary mouse keratinocytes with (1) recombinant Sema4C, a ligand for Plexin-B2^13^, (2) recombinant Sema4A or Sema4D, ligands for both Plexin-B1 and Plexin-B2^13^, or (3) recombinant Plexin-B1 or Plexin-B2, and measured the mRNA expression levels of the YAP target genes *ctgf* and *cyr61*. However, neither activation of semaphorin-plexin forward nor of reverse signaling was sufficient to suppress YAP target gene expression (Supplementary Fig. 4d, e). Interestingly, in Xenopus, Plexin-A1 has been described to interact *in trans* with Plexin-A1 on other cells, and this homophilic binding has been shown to depend on the presence of calcium ions^37^. We found that the localization of Plexin-B1 and Plexin-B2 to cell–cell contacts of primary mouse keratinocytes was stabilized in the presence of calcium (Fig. 4a and Supplementary Fig. 4f), which was intriguing given the strong calcium-dependency of YAP activity control by Plexin-B1/Plexin-B2 (Supplementary Fig. 3d–i). Structure–function experiments in a renal tubular epithelial cell line showed that this localization of Plexin-B2 to cell–cell contacts relied on its extracellular domain and was independent of its intracellular domain (Supplementary Fig. 4g). To test whether Plexin-B1 and Plexin-B2 mediate homophilic binding in the presence of calcium, we measured the adhesion of primary mouse keratinocytes to recombinant Plexin-B1 and Plexin-B2. Indeed, while control cells strongly adhered to recombinant Plexin-B1 and Plexin-B2, Plexin-B1/Plexin-B2-deficient cells adhered less efficiently (Fig. 4b). In contrast, we did not detect any binding of primary mouse keratinocytes to recombinant Sema4C (Supplementary Fig. 4h). Interestingly, recombinant Sema4A interfered with adhesion of primary mouse keratinocytes to recombinant Plexin-B1 (Supplementary Fig. 4i), suggesting that Sema4A could compete with homophilic plexin interactions. Taken together, these data show that Plexin-B1/Plexin-B2 localize to cell junctions and promote cell–cell adhesion via a homophilic-binding mechanism.

Similar to Plexin-B1/Plexin-B2, cadherins are calcium-dependent homophilic adhesion molecules, which act as mechanical integrators upstream of YAP^38–40^. We therefore asked whether plexins could mediate mechanosensation through the regulation of adherens junction organization and/or function. Indeed, while expression levels of E-cadherin did not change upon loss of Plexin-B1/Plexin-B2 (Supplementary Fig. 4j–o), we observed that the localization of E-cadherin to cell–cell contacts of dividing primary mouse keratinocytes in vitro (Fig. 4c) and of dividing epidermal stem cells in vivo (Fig. 4d, e) was strongly reduced by Plexin-B1/Plexin-B2-deficiency. Consequently, adhesion of Plexin-B1/Plexin-B2-deficient primary keratinocytes to E-cadherin was impaired (Fig. 4f). To assess the spatial relationship of Plexin-B2 and E-cadherin at cell–cell contacts of epidermal stem cells, we performed super-resolution structured illumination microscopy (SR-SIM) on whole-mount immunostainings of the embryonic epidermis at E15.5. Interestingly, Plexin-B2 and E-cadherin largely localized to different microdomains of the plasma membrane (Fig. 4g). Moreover, co-immunoprecipitation experiments confirmed that Plexin-B2 does not directly associate with E-cadherin complexes (Supplementary Fig. 4p). E-cadherin interacts with α-catenin, which is an upstream negative regulator of YAP and is critically involved in cell density sensing in the epidermis^30,39,41^. Fully consistent with the decrease in E-cadherin-based junctions upon loss of Plexin-B1/Plexin-B2, we found a diminished recruitment of α-catenin to junctions of dividing epidermal stem cells lacking Plexin-B1/Plexin-B2 (Fig. 4h, i). As further evidence for a role of Plexin-B1/Plexin-B2 in regulating mechanosensation at E-cadherin-based junctions, we observed decreased levels of a-18, the tension-sensitive epitope of α-catenin, in dividing epidermal stem cells deficient for Plexin-B1/Plexin-B2 (Fig. 4j, k). To directly assess whether Plexin-B1/Plexin-B2 mediate mechanosensation through regulation of cell junctions, we analyzed the alignment of keratinocyte monolayers relative to the direction of mechanical stretch, a response known to be junction-dependent^42^. While control cells robustly reoriented their major axes perpendicular to the direction of stretch, Plexin-B1/Plexin-B2 double-deficient cells entirely failed to mount a mechanoresponse (Fig. 4l, m and Supplementary Fig. 5). Collectively, these results indicate that Plexin-B1/Plexin-B2 stabilize adhesive cell junctions required for sensation of mechanical forces.

### Plexin-B1/Plexin-B2 regulate dynamic crowding-induced changes of cortical stiffness

**Fig. 5:**
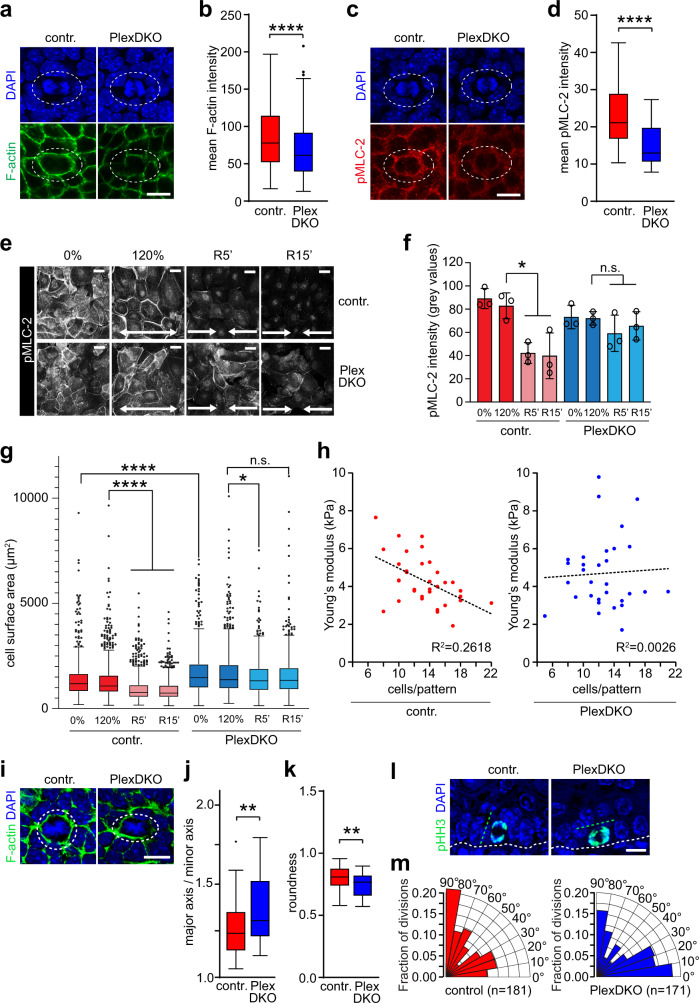
Plexin-B1/Plexin-B2 regulate dynamic crowding-induced changes of cortical stiffness. **a**, **c** Confocal images of the epidermal basal layer at E15.5 **a** stained with phalloidin (green) or **c** immunostained for anti-phosho-myosin light chain 2 (pMLC-2; Ser19; red). Cell divisions are contoured by dashed lines. Scale bar, 10 µm. **b**, **d** Quantification of **b** phalloidin fluorescence intensities and **d** pMLC-2 immunofluorescence intensities at cell–cell contacts between a dividing cell and its immediate neighbors (box plot with minimum, first quartile, median, third quartile and maximum). **b** control: *n* = 228 cell–cell contacts of 37 dividing cells from three mice, PlexDKO: *n* = 244 cell–cell contacts of 39 dividing cells from three mice. **d** control: *n* = 50 dividing cells from three mice, PlexDKO: *n* = 47 dividing cells from three mice. Two-sided Mann–Whitney *U* test for **b** and **d** *p* < 0.0001. **e**, **f** Representative pMLC-2 (Ser19) immunofluorescence images **e** and quantification **f** upon crowding (mean ± s.d., *n* = 3 independent experiments with >100 cells/condition/experiment; *p = 0.0322, Friedman/Dunn’s; scale bars 20 µm). **g** Quantification of cell surface areas after static 120% stretch and subsequent release (Tukey’s box and whiskers plot with minimum, first quartile, median, third quartile and maximum; *n* > 500 cells/condition pooled across three independent experiments; ****p < 0.0001, *p = 0.0131, Kruskal–Wallis/Dunn’s). **h** Force indentation spectroscopy of cell cortexes from primary mouse keratinocytes adhering on 100 µm circular micropatterns (control: *n* = 34, PlexDKO: *n* = 31 micropatterns pooled across three independe*n*t experiments). **i** Confocal images of the epidermal basal layer at E15.5 stained with phalloidin (green). Cell divisions are contoured by dashed lines. Blue: DAPI. Scale bar, 10 µm. **j**, **k** Quantification of shape anisotropy of basal cells in early mitosis (i.e. prophase to metaphase) (box plot with minimum, first quartile, median, third quartile and maximum; control: *n* = 44 cells from three mice, PlexDKO: *n* = 42 cells from three mice; **j** *p* = 0.0035, **k** *p* = 0.0033; two-sided Mann–Whitney *U* test). **l** Confocal images of the epidermal basal layer at E16.5 immunostained for phospho-histone H3 (green). White dashed line: plane of basal layer; green dashed line: axis of mitosis. Scale bar, 10 µm. **m** Radial histograms (rose plots) depicting the mitotic spindle angle of dividing control and Plexin-B1/Plexin-B2-deficient basal cells (control: *n* = 181 cells from six mice, PlexDKO: *n* = 171 cells from six mice; two-sided Mann–Whitney *U* test: *p* = 0.0187).

Cadherin-based cell junctions not only connect, via α-catenin, to the cortical actomyosin network, but also contribute to its biogenesis^39^. The cortical actomyosin network, in turn, generates cortical tension and promotes cadherin clustering and stability at cell–cell contact sites^39,40,43,44^. We therefore investigated whether Plexin-B1/Plexin-B2 impact on the organization and regulation of the actomyosin network. Indeed, cortical F-actin intensity at contact sites of dividing epidermal stem cells and their respective neighbor cells was less pronounced in the epidermis of Plexin-B1/Plexin-B2-deficient embryos as compared to control embryos (Fig. 5a, b). Moreover, the levels of phosphorylated myosin light chain 2 (pMLC-2), a marker for actomyosin contractility, were markedly reduced (Fig. 5c, d). In order to probe for a potential requirement of Plexin-B1/Plexin-B2 in the regulation of the actomyosin network by mechanical forces, we exposed control and Plexin-B1/Plexin-B2-knockout keratinocytes to static stretch and abrupt compression. These analyses revealed that, in contrast to control cells, Plexin-B1/Plexin-B2-deficient cells were unable to modulate the levels of pMLC-2 in response to crowding (Fig. 5e, f). We then asked whether this impaired modulation of pMLC-2 levels would correlate with an impaired regulation of cell surface area and cortical stiffness in response to crowding. When released from stretch, control primary mouse keratinocytes rapidly reduced their surface area (Fig. 5g). In striking contrast, keratinocytes lacking Plexin-B1/Plexin-B2 failed to respond by a surface area decrease (Fig. 5g). To examine the crowding-induced regulation of cortical stiffness, we performed atomic force microscopy (AFM)-mediated force indentation spectroscopy on micropatterns at different cell densities. While control cells, as expected, showed a linear inverse correlation of cortical elastic modulus and cell density, Plexin-B1/Plexin-B2-deficient cells exhibited no correlation (Fig. 5h). Taken together, these data demonstrate that Plexin-B1/Plexin-B2 are required for dynamic modulation of the actomyosin network, cell surface area, and cortical stiffness in response to lateral compression.

A number of studies have revealed that remodeling of the actomyosin network is required for mitotic cell rounding^45–48^. Moreover, B-plexins have been reported to induce cell rounding in COS-7^49^ and in HeLa cells^50^. Indeed, we observed an impaired rounding of Plexin-B1/Plexin-B2-deficient epidermal stem cells in mitosis (Fig. 5i–k). In addition to regulating mitotic cell rounding, the cortical actomyosin network has also been shown to control mitotic spindle orientation, which plays a critical role in epidermal stem cell biology^45,47,48,51–53^. Moreover, plexins have been identified as critical determinants of mitotic spindle orientation in neuroepithelial progenitors and renal tubular epithelial cells^23,54^. In line with this, we found mitotic spindle orientation of embryonic epidermal stem cells to be perturbed by loss of Plexin-B1/Plexin-B2, with Plexin-B1/Plexin-B2-deficient stem cells dividing at lower angles, i.e. more parallel to the basement membrane than cells of control embryos (Fig. 5l, m). Taken together, these data further corroborate the function of Plexin-B1/Plexin-B2 in modulation of actomyosin-dependent processes in epidermal stem cells.

### Plexin-B1/Plexin-B2 control YAP activity and cell proliferation in basal cell carcinoma

Next, we asked whether Plexin-B1/Plexin-B2-mediated mechanosensation of proliferation-induced tissue crowding could also be relevant in skin disease. Consistent with the low rate of stem cell divisions in the adult murine epidermis (Fig. 1a, b)^19,20^, genetic inactivation of Plexin-B1 and Plexin-B2 at adult stages using a tamoxifen-inducible K14-CreERT line (genotype K14-CreERT;*plxnb1*^flox/flox^;*plxnb2*^flox/flox^; Supplementary Fig. 6a) did not result in any morphological abnormalities (Supplementary Fig. 6b–g). We hypothesized that an experimental triggering of cell divisions in the adult by topic application of a chemical mitogen to the skin would again disclose the requirement of Plexin-B1/Plexin-B2 for the inhibitory mechanical feedback on cell proliferation. Indeed, treatment of Plexin-B1/Plexin-B2-deficient mice with the chemical mitogen, the phorbol ester TPA (Supplementary Fig. 7a) resulted in higher proliferation rates (Supplementary Fig. 7b, c) and more pronounced epidermal thickening (Supplementary Fig. 7d, e) than in control mice. Fully in line with the function of Plexin-B1/Plexin-B2 as mechanosensitive suppressors of YAP activity during skin development, YAP was also found to be disinhibited in the TPA-treated skin of adult Plexin-B1/Plexin-B2 knockout mice (Supplementary Fig. 7f, g).

**Fig. 6:**
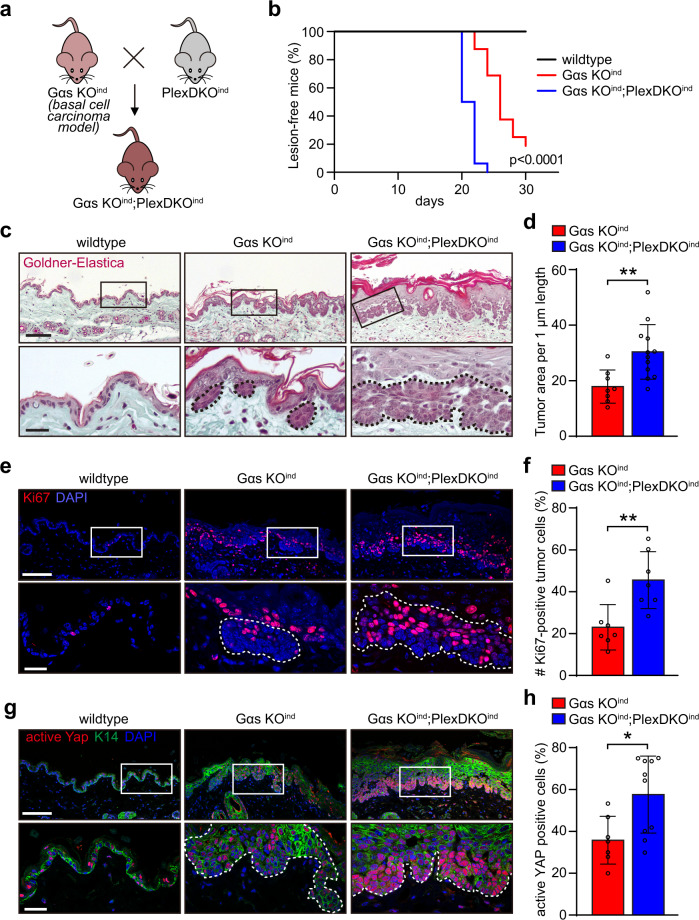
Plexin-B1/Plexin-B2 control YAP activity and cell proliferation in basal cell carcinoma. **a** Schematic illustration of the generation of epidermis-specific tamoxifen-inducible Gα_s_/Plexin-B1/Plexin-B2 triple-deficient mice. **b** Kaplan–Meier curves representing the percentage of lesion-free mice. Time point “0” indicates the start of tamoxifen treatment (wildtype mice: *n* = 3, Gαs KO^ind^ mice: *n* = 16, Gαs KO^ind^;PlexDKO^ind^ mice: *n* = 16; *p* < 0.0001; Mantel–Cox test for Gαs KO^ind^ compared to Gαs KO^ind^;PlexDKO^ind^). **c–h** Histological/immunofluorescence stainings (left panels) and respective quantifications (right panels) of adult murine skin of mice with the indicated genotypes 30 days after treatment with tamoxifen. Boxed areas are magnified in the lower rows. Tumors are marked by dashed lines. **c** Goldner-Elastica stain of histological sections. Scale bar, 100 µm (upper row), 25 µm (lower row). **d** Quantification of tumor areas (mean ± s.d.; Gαs KO^ind^ mice: *n* = 8, Gαs KO^i*n*d^;PlexDKO^ind^ mice: *n* = 12; *p* = 0.0049; two-sided unpaired *t*-test). **e** Confocal images of immunostainings using an anti-Ki67 antibody (red). Scale bar, 100 µm (upper row), 25 µm (lower row). **f** Quantification of Ki67-positive cells (mean ± s.d.; Gαs KO^ind^ mice: *n* = 7, Gαs KO^i*n*d^;PlexDKO^ind^ mice: *n* = 7; *p* = 0.0049; two-sided unpaired *t*-test). **g** Confocal images of immunostainings using anti-active YAP (red) and anti-K14 antibodies (green). Scale bar, 100 µm (upper row), 25 µm (lower row). **h** Quantification of cells positive for active YAP (mean ± s.d.; Gαs KO^ind^ mice: *n* = 7, Gαs KO^i*n*d^;PlexDKO^ind^ mice: *n* = 10; *p* = 0.0143; two-sided unpaired *t*-test).

To assess the potential pathophysiological relevance of mechanosensation through Plexin-B1/Plexin-B2 in disease, we next analyzed the role of Plexin-B1/Plexin-B2 in skin tumors. To do so, we employed two genetic mouse models for basal cell carcinoma, the most common type of skin cancer^55^. In these mouse models, epidermal stem cell proliferation and tumor formation is driven by tamoxifen-inducible epidermis-specific inactivation of the tumor suppressors Gαs or Patched1, the most frequently mutated gene in human basal cell carcinoma^56^ (Fig. 6a and Supplementary Fig. 8a). Interestingly, in both genetic models, mice lacking Plexin-B1 and Plexin-B2 displayed a higher incidence (Fig. 6b and Supplementary Fig. 8b) and larger sizes of tumors (Fig. 6c, d and Supplementary Fig. 8c, d) than the respective control mice. Furthermore, disruption of Plexin-B1 and Plexin-B2 resulted in a strong increase in cell proliferation (Fig. 6e, f and Supplementary Fig. 8e, f) and a marked up-regulation of YAP activity (Fig. 6g, h and Supplementary Fig. 8g, h). In summary, these data indicate that Plexin-B1/Plexin-B2 limit YAP activity and proliferative capacity not only during embryonic development but also in skin cancer in mice.

**Fig. 7:**
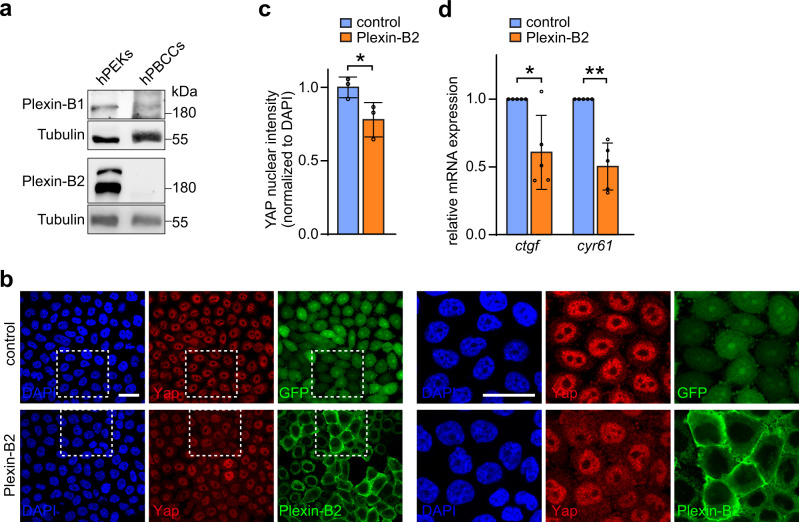
B-plexins suppress YAP activity in human basal cell carcinoma cells. **a** Human primary epidermal keratinocytes (hPEKS) and human primary basal cell carcinoma cells (hPBCCs) were lysed, and proteins were detected using the indicated antibodies. **b**, **c** hPBCCs engineered to express GFP (“control”) or myc-tagged Plexin-B2 (“Plexin-B2”) and cultured at dense conditions were immunostained for YAP (red) and myc (green). Blue: DAPI. Representative confocal images are shown in **b** with dashed boxed areas depicted in the right panel. A quantification of YAP nuclear intensity is shown in **c** (mean ± s.d.; *n* = 3 independent experiments; *p* = 0.0495; two-sided unpaired *t*-test). **d** hPBCCs were engineered to express GFP (“control”) or myc-tagged Plexin-B2 (“Plexin-B2”), and cultured at dense conditions. Relative mRNA expression levels of the YAP target genes *ctgf* and *cyr61* were determined by quantitative RT-PCR (mean ± s.d.; *n* = 5 independent experiments; *p* = 0.0123 for *ctgf*, *p* = 0.0002 for *cyr61*; two-sided unpaired *t*-test).

To investigate a potential role of Plexin-B1 and Plexin-B2 for basal cell carcinoma progression in humans, we next studied primary human basal cell carcinoma cells. Interestingly, in these cells, Plexin-B2 protein expression was almost lost and Plexin-B1 protein expression was markedly lower than in benign human primary keratinocytes (Fig. 7a), supporting the concept that loss of Plexin-B1/Plexin-B2 could provide a mechanistic basis for basal cell carcinoma progression also in humans. The strongly reduced expression levels of Plexin-B1 and Plexin-B2 correlated with an impaired ability of the primary human cancer cells to exclude YAP from the nucleus at high cell densities (Supplementary Fig. 9a, b). To test for a functional role of B-plexins in the control of YAP activity in human primary basal cell carcinoma cells, we engineered cells to re-express Plexin-B2 (Supplementary Fig. 9c). Indeed, Plexin-B2-expressing primary human basal cell carcinoma cells displayed less nuclear YAP and lower mRNA expression levels of YAP target genes than GFP-expressing control cells (Fig. 7b–d). In summary, these data suggest that the B-plexin-dependent mechanochemical feedback could also be relevant in basal cell carcinomas in human patients.

## Discussion

Cell divisions within the jammed basal layer of the mouse embryonic epidermis result in crowding and mechanical stress anisotropy. Our study is in line with a concept, in which crowding acts as a central checkpoint in the control of epidermal morphogenesis by triggering mechanisms to reinstate stem cell density. These mechanisms include differentiation and extrusion of differentiating cells from the stem cell layer by upward efflux^18,57–59^, as well as suppression of stem cell divisions by mechanical feedback^41^. Our data now identify the transmembrane receptors Plexin-B1 and Plexin-B2 as key to this feedback by coupling mechanosensation of cell density to the biochemical control of YAP activity and cell proliferation. Our work demonstrates that the Plexin-B1/Plexin-B2-mediated mechanoresponse to crowding is particularly relevant for proper morphogenesis of the early embryonic epidermis where proliferation rates are high. The sharp decrease of Plexin-B1 and Plexin-B2 expression levels at late embryonic stages and, in particular, the absence of epidermal abnormalities after induction of Plexin-B1/Plexin-B2 deficiency in adult mice indicate that—in contrast to early development—the mechanosensation of crowding does not coordinate epidermal homeostasis when proliferation rates are low. This is consistent with previous studies showing that in the adult interfollicular epidermis, stem cells divide only upon demand to replace delaminating cells, and thus crowding does not occur^10,60^. Intriguingly, we found that mechanosensation through Plexin-B1/Plexin-B2 regains biological relevance in the adult murine epidermis for the suppression of cancer cell proliferation. In line with this, we observed that expression levels of Plexin-B1 and Plexin-B2 in primary human basal cell carcinoma cells were low, and that re-expression of Plexin-B2 was sufficient to suppress YAP activity. Taken together, these data strongly suggest that loss of Plexin-B1/Plexin-B2 could represent a mechanistic basis for the loss of contact inhibition, a hallmark of cancer that has remained poorly understood on a molecular level^61,62^. In support of this concept, Plexin-B1 has been shown to also suppress proliferation of human renal adenocarcinoma^63^ and melanoma cell lines^64^, and to inhibit primary tumor growth of human melanoma cells in a xenograft mouse model^64^. Moreover, in human patients, Plexin-B1 is down-regulated in renal cell carcinoma^63^ and melanoma^65^, and low expression levels of Plexin-B1 correlate with bad prognosis in particular subsets of breast cancer^66–68^. It has to be noted, however, that the functions of plexins in cancer are highly context-dependent, and that in other settings, plexins can also promote tumor progression^13,14,69,70^. On a molecular level, this functional versatility of plexins is reflected by their association with multiple co-receptors and signaling molecules that significantly impact on plexin function^71,72^.

The extracellular domain of several plexins has been shown to adopt different conformations ranging from a nearly closed, ring-like form to a more-open, chair-like form^17,73,74^. Recently, a particular plexin, Plexin-D1, has been reported to sense shear stress-induced mechanical forces in endothelial cells^17^. This function in mechanosensation required the opening of the ring-like conformation of the Plexin-D1 extracellular domain, but was independent of the binding of semaphorins^17^. This is in line with our findings in epithelial cells, showing that activation of Plexin-B1 and Plexin-B2 by semaphorins is not sufficient to regulate the activity of the mechanotransducer YAP. The similar structures of the extracellular domains of plexins^13,73^ suggest that the mechanism of mechanosensation by semaphorin-independent conformational changes could be shared by different plexins including Plexin-B1 and Plexin-B2.

In addition to B-plexins, several other proteins have been described to play a role in mechanosensation and mechanotransduction in the epidermis. These include the mechanosensitive ion channels Piezo1 in keratinocytes^75^ and Piezo2 in Merkel cells^76,77^, E-cadherin^38–40,78,79^, and integrins^29,80^. E-cadherin molecules organize into microclusters, the formation of which requires homophilic binding at cell–cell contacts^81^. Our data show that B-plexins aggregate into similar clusters, which also depend on homophilic interactions. Albeit spatially separated from E-cadherin aggregates, these B-plexin microclusters are required for proper E-cadherin localization at the plasma membrane of epidermal stem cells.

As opposed to B-plexins, which we have identified as suppressors of YAP activity, integrins promote YAP activity in the epidermis via Src and FAK signaling^29^. Interestingly, Plexin-B1 has been shown to inhibit integrins and FAK in several cell lines in a semaphorin-dependent manner^49,82^. It is therefore conceivable that B-plexins—in addition to their ligand-independent role in mechanosensation—also mediate a semaphorin-induced inhibition of integrin activity in the epidermis, and thereby suppress a functionally antagonistic mechanosensitive pathway.

In summary, our work uncovers a critical requirement for Plexin-B1 and Plexin-B2 in the control of epidermal stem cell proliferation by mechanical forces. Whether the function of Plexin-B1 and Plexin-B2 in mechanosensation is specific for crowding-induced compression remains open to future research; however, it seems more likely that they could respond to a wider spectrum of different mechanical stimuli, a property that is known from other types of mechanosensors such as mechanosensitive ion channels^83^. Given the wide expression of Plexin-B1 and Plexin-B2 in epithelial and non-epithelial cells both during development as well as in the adult^13,84,85^, it is tempting to speculate that mechanosensation by Plexin-B1 and Plexin-B2 could be instrumental in physiology and pathophysiology of multiple tissues.

## Methods

### Mice

To generate mice lacking Plexin-B1 and Plexin-B2 specifically in the epidermis, mice carrying conditional alleles of Plexin-B1^86^ and Plexin-B2^86^ were crossed with mice expressing Cre constitutively under the control of the keratin 14 (K14) promoter^24^, or with mice expressing CreERT under the control of the K14 promoter^87^ (Tg(KRT14-cre/ERT)20Efu/J, The Jackson Laboratory, Stock No. 005107). Mice carrying floxed alleles of Gα_s_^88^ or of Patched1^89^ have been described previously. All mice used in this study were on a C57BL/6 genetic background. Mice were housed under a 12-h light–dark cycle with free access to food and water, and under specific pathogen-free conditions. All procedures were performed in accordance with German Animal Welfare legislation, and have been approved by the Regierungspräsidium Darmstadt and Giessen.

### Antibodies

The following primary antibodies were used: guinea pig polyclonal anti-keratin 14 (1:200, Progen, cat. no. GP-CK14), rabbit polyclonal anti-keratin 14 (1:200, Biolegend, cat. no. 905301), rabbit polyclonal anti-keratin 10 (1:200, Biolegend, cat. no. 905401), rabbit polyclonal anti-Ki67 (1:200, Abcam, cat. no. ab15580), biotinylated monoclonal anti-BrdU (1:100, Biolegend, cat. no. 339810), rabbit polyclonal anti-phospho-histone H3 (1:200, Cell Signaling, cat. no. 9701), mouse monoclonal anti-Plexin-B1 (for Western Blot; 1:500, Santa Cruz, cat. no. sc-28372), armenian hamster monoclonal anti-mouse Plexin-B2 (for immunostainings; 1:200, eBioscience, cat. no. 14-5665-85), sheep polyclonal anti-mouse Plexin-B2 (for Western Blot; 1:500, R&D Systems, cat. no. AF6836), sheep polyclonal anti-human Plexin-B2 (for Western Blot; 1:500, R&D Systems, cat. no. AF5329), rabbit monoclonal anti-E-cadherin (1:200, Cell Signaling, cat. no. 3195), rabbit polyclonal anti-α-catenin (1:200, Invitrogen, cat. no. 71-1200), anti-α-catenin a-18 (for immunostainings on elastomers; 1:10,000^90^), mouse monoclonal anti-β-catenin (1:1000; BD Bioscience, cat. no. 610153), rabbit monoclonal anti-cleaved Notch1 (NICD, 1:100, Cell Signaling, cat. no. 4147), rabbit monoclonal anti-Yap antibody (1:200, Cell Signaling, cat. no. 14074), mouse monoclonal anti-Yap (for immunostainings on elastomers; 1:300, Santa Cruz; sc-101199), rabbit monoclonal anti-active Yap (1:200, Abcam, cat. no. ab205270), rabbit polyclonal anti-phospho-myosin light chain 2 (Ser19) (1:200, Cell Signaling, cat. no. 3671), rabbit polyclonal anti-phospho-myosin light chain 2 (Thr18/Ser19) (for immunostainings on elastomers; 1:200, Cell Signaling; cat. no. #3674), rabbit polyclonal anti-c-myc-Peroxidase (for Western Blot; 1:2000, Sigma, cat. no. A5598), mouse monoclonal anti-myc (for immunostainings; 1:500, Cell Signaling, cat. no. 2276), rabbit monoclonal anti-α-tubulin (1:1000, Cell Signaling, cat. no. 2125). An anti-Plexin-B1 antibody was raised against the extracellular domain of Plexin-B1.

The following secondary antibodies were used: Cy3-conjugated anti-armenian hamster (1:200; Jackson ImmunoResearch, cat. no. 127-165-160), AlexaFluor 488-conjugated anti-guinea pig (1:200; Invitrogen, cat. no. A11073), AlexaFluor 488-conjugated anti-rabbit (1:200; Invitrogen, cat. no. R37118), AlexaFluor 488-conjugated anti-rat (1:200; Invitrogen, cat. no. A-11006), AlexaFluor 555-conjugated anti-rabbit (1:200; Invitrogen, cat. no. A21429), AlexaFluor 555-conjugated anti-mouse (1:200; Invitrogen, cat. no. A21424), AlexaFluor 488-conjugated anti-mouse (1:200; Invitrogen, cat. no. A11029), HRP-conjugated anti-sheep (1:5000, Invitrogen, cat. no. A16041), HRP-conjugated anti-rabbit (1:5000, BioRad, cat. no. 1706515), and HRP-conjugated anti-mouse (1:5000, VWR, cat. no. NA9310).

The following reagents were used: AlexaFluor 488-conjugated phalloidin (1:500, Invitrogen, cat. no. A12379) and AlexaFluor 647-conjugated phalloidin (1:500, Invitrogen, cat. no. A22287).

Uncropped western blots can be found in the Source data file.

### Recombinant proteins

Recombinant human Sema4A (cat. no. ab182683) was purchased from Abcam. Recombinant mouse Sema4C (cat. no. 8394-S4-050), recombinant mouse Sema4D (cat. no. 5235-S4B), recombinant mouse Plexin-B2 (cat. no. 6836-PB-050), and recombinant mouse E-cadherin (cat. no. 8875-EC-050) were purchased from R&D Systems. A fragment of human Plexin-B1 cDNA (encoding amino acids 1-535) was cloned into the pcDNA5 vector using HindIII (R0104S, NEB) and XhoI (R0146S, NEB) restriction sites with addition of a C-terminal 6xHis tag (KHHHHHH). The expression plasmid was transfected into Expi293F cells (Invitrogen) according to the manufacturer’s instructions. The supernatant containing secreted Plexin-B1 (amino acids 20-535)-6xHis was collected after 7 days and purified using a two-step purification protocol developed at Medical Research Council Technology (MRC-T). Briefly, overexpressed protein was captured on an Excel HisTrap (GE) column in 20 mM HEPES, pH 8.0, 0.3 M NaCl, and 10 mM imidazole buffer. After washing the column with 10 column volumes of washing buffer (20 mM HEPES, pH 8.0, 0.3 M NaCl and 20 mM imidazole), the protein was eluted with elution buffer (20 mM HEPES, pH 8.0, 0.3 M NaCl and 250 mM imidazole), followed by gel filtration chromatography using a Superdex 200 16/60 column equilibrated with PBS. Collected fractions were analyzed on SDS–PAGE, pooled, quantified by NanoDrop, aliquoted and flash-frozen for storage at −80 °C.

### Quantitative RT-PCR

Total RNA from murine skin, murine epidermis, and primary mouse keratinocytes was isolated using Direct-zol RNA Microprep Kits (Zymo) according to the manufacturer’s instructions. Genomic DNA was removed by on-column DNAse digestion, and cDNA was generated by reverse transcription using the RevertAid First Strand cDNA Synthesis Kit (ThermoFisher). qRT-PCR was performed with SYBR Green Supermix (BioRad) in a Real Time Quantitative Thermal Cycler (BioRad). For absolute quantifications, mouse genomic DNA was used as a standard. For relative quantifications, gene expression changes were normalized to *gapdh* or *actb* expression. Complete lists of all primers used are supplied as Supplementary Tables 1 and 2.

### Cell lines

MDCK cells, a canine renal tubular epithelial cell line, were cultured in MEM supplemented with 10% FBS^23^.

### Primary cells

Primary mouse keratinocytes were isolated and cultured essentially as described^78^. Briefly, the skin of newborn mice was harvested using sterile forceps, and incubated in 1 ml dispase (5 mg/ml) in culture medium (CnT-07; Cellntec) in a 2-ml-microcentrifuge tube over night at 4 °C. The skin was then transferred to a 10-cm-Petri dish, and washed with PBS. Using forceps, the epidermal sheet was separated from the dermis, transferred with the basal layer facing downwards onto 500 µl of accutase (Cellntec) in a 6-cm-Petri dish, and incubated for 30 min at RT. Keratinocytes were washed out of the epidermal basal layer using 2 ml of culture medium, collected by centrifugation, and seeded onto collagen type 1 (30 µg/ml, Biochrom, cat no. L7213)-coated 10-cm-Petri dishes. Primary mouse keratinocytes were kept in culture medium (CnT-07; Cellntec) at 37 °C and 5% CO_2_. Only low passage cells (max. 2 passages) were used for experiments. For the analysis of semaphorin-plexin forward and reverse signaling, primary mouse keratinocytes were incubated with recombinant proteins (25 nM) for 8 h prior to RNA isolation.

Human primary epidermal keratinocytes (hPEKs) were purchased from CellnTec and were grown in keratinocyte growth medium (cat. no. CnT-PR; CellnTec). Human primary basal cell carcinoma cells (hPBCCs) were purchased from Celprogen and were grown in human basal cell carcinoma cell line complete media with serum (cat. no. M77015-08S, Celprogen).

### Lentiviral and retroviral transduction

To generate an E-cadherin-mRuby lentiviral construct, the human cDNA of E-cadherin was amplified by PCR and fused to the mRuby2 sequence using overlap/extension PCR. The fused cDNA was ligated into pWPXL via Mlu1 and Spe1 restriction sites. Triple-myc-tagged full-length mouse Plexin-B2^91^ was ligated into the pENTR1A entry vector using SalI/NotI restriction enzymes. By recombination using the Gateway LR Clonase (Invitrogen), the cDNA was then inserted into the lentiviral expression vector pINDUCER20^92^, which allows for doxycycline-inducible expression of triple-myc-tagged full-length mouse Plexin-B2. To generate a respective control plasmid, GFP was ligated into the pENTR1A entry vector using NcoI/NotI restriction enzymes, followed by recombination into pINDUCER20 using the Gateway LR Clonase (Invitrogen). For lentivirus production, HEK293T cells were cotransfected (using the calcium phosphate method) with the packaging plasmids psPAX2 and pMD2.G together with the aforementioned lentiviral constructs. After 48 h, supernatants containing viral particles were harvested, filtered, and used to transduce primary cells. To select for transduced hPBCCs, cells were treated with puromycin (10 µg/ml) for 5 days. To induce the expression of GFP or Plexin-B2, respectively, cells were treated with doxycycline (1 µg/ml) for 24 h.

To generate MDCK cells stably expressing Plexin-B2-EGFP or expressing a Plexin-B2 mutant, which lacks the intracellular domain (Plexin-B2ΔIC-EGFP), the murine cDNAs encoding triple-myc-tagged wildtype Plexin-B2 (amino acids 20-1842)^91^ or triple-myc-tagged truncated Plexin-B2 (amino acids 20-1227) were cloned into pEGFP-N1 using AfeI and SalI restriction sites. The fused cDNAs encoding Plexin-B2 and EGFP were ligated into pLNCX2 using NotI. For retrovirus production, PT67 cells were transfected with the aforementioned retroviral constructs. After 48 h, supernatants containing viral particles were harvested, filtered, and used to transduce MDCK cells (in the presence of polybrene; 8 µg/ml).

### H&E and Goldner-Elastica staining

Hematoxylin/Eosin (H&E) and Goldner-Elastica stainings were performed on paraffin-embedded sections using standard laboratory protocols.

### Immunofluorescence staining

For whole-mount immunofluorescence stainings, the epidermis was harvested from the dorsal skin of E15.5 embryos using forceps, fixed in 4% PFA for 30 min, washed twice in 50 mM glycine in PBS, followed by washing twice in PBS. After permeabilization with PBST (0.5% Triton X-100 in PBS) for 20 min and blocking with 4% FCS in PBST for at least 1 h, the epidermis was incubated with primary antibodies (diluted in 2% FCS in PBST) at 4 °C overnight. After three times washing in PBST, samples were incubated with secondary antibodies and DAPI for 3 h, and washed. All processing of samples was done in 12-well or 96-well plates. Samples were then mounted on glass slides using Fluoromount (Dako). Images were taken using a confocal laser scanning microscope (Zeiss LSM 700).

For immunofluorescence stainings of paraffin-embedded sections, tissues (dorsal skin of mouse embryos or adult mouse skin/tumor tissue) were fixed in 4% PFA at 4 °C overnight, embedded in paraffin according to standard laboratory protocols, sectioned at 5 μm, and mounted on glass slides. Antigen retrieval was done by boiling in a steam pressure pot in 10 mM sodium citrate buffer pH 6.0 for 10 min. Sections were permeabilized with PBST (0.2% Triton X-100 in PBS) for 10 min, blocked with 4% FCS in PBST for at least 1 h and incubated with primary antibodies (diluted in 2% FCS in PBST) at 4 °C overnight, washed with PBS, and incubated with secondary antibodies and DAPI at room temperature for 1 h. After three times washing in PBS, sections were mounted with Fluoromount (Dako). Images were taken using a confocal laser scanning microscope (Zeiss LSM 700).

For immunofluorescence staining of cryotome sections, dorsal skin of mouse embryos or adult mouse skin were fixed in 0.2% PFA overnight at 4 °C, followed by one day of incubation in 30% sucrose (in PBS). Tissues were embedded in OCT medium, frozen on dry ice, and sectioned using a cryotome (thickness of sections 25 μm), and mounted on glass slides. Sections were postfixed in 4% PFA at 4 °C for 30 min, washed twice in 50 mM glycine in PBS, permeabilized with PBST (0.2% Triton X-100 in PBS), blocked with 4% FCS in PBST for at least 1 h and incubated with primary antibodies (diluted in 2% FCS in PBST) overnight at 4 °C. Sections were washed three times in PBS, incubated with secondary antibodies and DAPI at room temperature for 1 h. After three times washing in PBS, sections were mounted with Fluoromount (Dako). Images were taken using a confocal laser scanning microscope (Zeiss LSM 700).

For immunofluorescence stainings of primay mouse keratinocytes, keratinocytes (seeded into μ-Slide eight-well (ibidi) or onto micropatterns) were fixed in 4% PFA for 30 min, washed three times with PBS, permeabilized with PBST (0.2% Triton X-100 in PBS) for 10 min, blocked with 4% FCS in PBST for at least 1 h, incubated with primary antibodies (diluted in 2% FCS in PBST) at 4 °C overnight, washed with PBS and incubated with secondary antibodies and DAPI at room temperature for 1 h. After three times washing in PBS, cells were mounted with Fluoromount (Dako). Images were taken using a confocal laser scanning microscope (Zeiss LSM 700).

For immunostainings on silicone elastomers, cells were fixed in 4% PFA for 10 min room temperature, permeabilized with 0.3% Triton X-100 in PBS, and blocked in 5% bovine serum albumin (BSA). Samples were subsequently incubated overnight in primary antibody in 1% BSA/0.3% Triton X-100/PBS, followed by washing in PBS and incubation in secondary antibody in 1% BSA/0.3% Triton X-100/PBS. Finally, samples were mounted in Elvanol. Images were collected by laser scanning confocal microscopy (SP8X; Leica) with Leica Application Suite software (LAS X version 2.0.0.14332), using ×40 immersion objectives. Images were acquired at room temperature using sequential scanning of frames of 1 µm-thick confocal planes (pinhole 1) after which 10 planes encompassing complete cell nuclei were projected as a maximum intensity confocal stack. Images were collected with the same settings for all samples within an experiment.

### BrdU labeling

Pregnant females were injected i.p. with 200 µl of BrdU (Sigma, cat. no. B5002; 7.5 mg/ml in PBS) 2 h prior to collection of embryos. BrdU incorporation was detected by immunohistochemical staining using an anti-BrdU antibody.

### Immunohistochemical staining

For immunohistochemical stainings of paraffin-embedded sections, tissues were fixed in 4% PFA at 4 °C overnight, embedded in paraffin according to standard laboratory protocols, sectioned at 5 μm, and mounted on glass slides. Antigen retrieval was done by boiling in a steam pressure pot in 10 mM sodium citrate buffer pH 6.0 for 10 min. For immunohistochemical staining of NICD, sections were incubated with BLOXALL (ImmPRESS Excel Staining Kit; Vector laboratories, cat. no. MP-7601) to inactivate endogenous peroxidases for 10 min, washed in PBS, permeabilized with PBST (0.2% Triton X-100 in PBS) for 10 min, blocked with 4% FCS in PBST for at least 1 h, and incubated with anti-NICD antibody (diluted in 2% FCS in PBST) at 4 °C overnight, washed with PBS, followed by usage of the ImmPRESS Excel Staining Kit (Vector laboratories, cat. no. MP-7601). Sections were then dehydrated and mounted with Pertex. For immunohistochemical staining of BrdU, sections were incubated in 0.3% H_2_O_2_ in methanol for 20 min, washed in PBS, and incubated in 2 M HCl at 37 °C for 30 min. Sections were then washed with PBST (0.2% Triton X-100 in PBS), blocked with 4% FCS in PBST for at least 1 h, and incubated with anti-BrdU antibody (diluted in 2% FCS in PBST) at 4 °C overnight. After washing with PBST, the ABC HRP Kit (Vector laboratories, cat. no. PK-4001) and the DAB Peroxidase (HRP) Substrate Kit (Vector laboratories, cat. no. SK-4100) were used according to the manufacturer’s instructions. Sections were then dehydrated and mounted with Pertex.

### TUNEL assay

TUNEL assays were performed on paraffin-embedded sections of the dorsal skin of embryos using the DeadEnd Fluorometric TUNEL System (Promega, cat. no. G3250) according to the manufacturer’s instructions.

### Immunoprecipitation

For protein immunoprecipitation, primary mouse keratinocytes were lysed in ice-cold buffer containing 50 mM Tris pH 7.6, 150 mM NaCl, 1% NP-40, 1.8 mM CaCl_2_ with complete EDTA-free Protease Inhibitor cocktail (Roche). Lysates were cleared by centrifugation, and supernatants were incubated with 2 µg anti-E-cadherin antibody in the presence of Protein G PLUS Agarose beads (Santa Cruz Biotechnology). Agarose beads were washed four times with lysis buffer, and immunocomplexes were eluted in Laemmli buffer. Samples were then subjected to immunoblot analysis.

### Surface biotinylation assay

Confluent primary mouse keratinocytes in 10-cm Petri dishes were washed twice with ice-cold PBS followed by incubation with 8 ml of Biotin-NHS (Sigma, cat. no. 203112) in PBS (0.25 mg/ml) for 10 min at 4 °C with gentle shaking. Afterwards, the reaction was quenched with the addition of 6 ml Tris–HCl (50 mM, pH 7,4). Cells were scraped off the dish, collected in a 50 ml tube, pelleted by centrifugation at 4 °C, and washed three times with ice-cold PBS. The pellet was resuspended in 800 µl of RIPA buffer (50 mM Tris–HCl, 300 mM NaCl, 1 mM EDTA, 1% Triton X-100, 0.25% sodium deoxycholate; 0.1% SDS, 5 mM MgCl_2_; pH 7.4) containing cOmplete Protease Inhibitor Cocktail (Roche, cat. no. 4693132001), and sonicated three times. After centrifugation at 4 °C, supernatants were incubated with Pierce Streptavidin Agarose (ThermoFisher, cat. no. 20353) for 90 min at 4 °C. Agarose beads were washed four times with ice-cold lysis buffer, and boiled in Laemmli buffer before analysis on SDS–PAGE followed by a Western blotting.

### Fluorescence in situ hybridization (FISH)

Fluorescence in situ hybridizations (FISH) were done on paraffin-embedded sections (5 µm) using the RNAscope system (ACD). The following probes were used: Mm-CTGF (ACD, cat. no. 314541), Positive Control Mm-Ppib (ACD, cat. no. 313911), Negative Control DapB (ACD, cat.no. 310043). The following kit was used: RNAscope 2.5 HD Detection Reagents-RED (ACD, cat. no. 322360). FISH was combined with immunofluorescence staining as described above.

### Live cell imaging

Live cell imaging of primary mouse keratinocytes was performed using a Thunder Imaging System (Leica) at 37 °C in a CO_2_ humidified incubation chamber in cell culture medium (CnT-07, Cellntec).

### Adhesion assay

Adhesion assays were performed essentially as described^37^. Mixed Cellulose Ester 96-well plates (Millipore, cat. no. MSHAS4510) were coated without or with recombinant proteins (5 µg/ml) at 4 °C overnight. Wells were then blocked with 5% bovine serum albumin (BSA) for 1 h at room temperature. After washing with PBS, 1 × 10^4^ primary mouse kerationocytes (in culture medium with 1.8 mM calcium) were added per well, and allowed to adhere for 30 min at 37 °C and 5% CO_2_. Afterwards, non-adherent cells were removed by washing twice with PBS. For some control wells, non-adherent cells were not removed by washing and all seeded cells remained in the well. The CellTiter-Glo Luminescent Cell Viability Assay (Promega, cat. no. G7572) was used according to the manufacturer’s protocol. The following three conditions were analyzed: (condition 1) Primary keratinocytes were added to uncoated wells. Wells were not washed and non-adherent cells were not removed, i.e. all seeded cells remained in the well; (condition 2) Primary keratinocytes were added to uncoated wells. Wells were washed and non-adherent cells were removed; (condition 3) Primary keratinocytes were added to wells coated with recombinant proteins. Wells were washed and non-adherent cells were removed; specific adhesion of primary keratinocytes (in %) was then calculated by the following formula: specific adhesion = (RLU condition 3−RLU condition 2)/RLU condition 1 = (RLU specifically adhered cells minus RLU non-specifically adhered cells) divided by RLU total cells =

$$\frac{{{\mathrm{RLU}}\,{\mathrm{of}}\,{\mathrm{coated}}\,{\mathrm{well}}\,{\mathrm{after}}\,{\mathrm{removal}}\,{\mathrm{of}}\,{\mathrm{non}}\,{\mathrm{adherent}}\,{\mathrm{cells}} - {\mathrm{RLU}}\,{\mathrm{of}}\,{\mathrm{non}}\,{\mathrm{coated}}\,{\mathrm{well}}\,{\mathrm{after}}\,{\mathrm{removal}}\,{\mathrm{of}}\,{\mathrm{non}}\,{\mathrm{adherent}}\,{\mathrm{cells}}}}{{{\mathrm{RLU}}\,{\mathrm{of}}\,{\mathrm{non}}\,{\mathrm{coated}}\,{\mathrm{well}}\,{\mathrm{without}}\,{\mathrm{removal}}\,{\mathrm{of}}\,{\mathrm{non}}\,{\mathrm{adherent}}\,{\mathrm{cells}}}}$$

### Pharmacological treatments

Mice at the age 6 weeks were injected with tamoxifen (1 mg; i.p.) for 5 consecutive days. One week after the start of tamoxifen injections, the dorsal hair was shaved. Two days later, DMBA (25 µg = 97.5 nmol, dissolved in acetone) was applied to the shaved back skin. Starting 2 weeks after the treatment with DMBA, 12-O-Tetradecanoylphorbol-13-acetate (TPA; 4 µg = 6.5 nmol, dissolved in acetone) was applied to the same site twice per week for a total of 14 weeks. The analyzed genotypes were “contr.” (genotype *plxnb1*^flox/flox^;*plxnb2*^flox/flox^) and “PlexDKO^ind”^ (genotype K14-CreERT;*plxnb1*^flox/flox^;*plxnb2*^flox/flox^). Mice were regularly monitored for the appearance of skin lesions, and did not develop papillomas. Only female mice were examined.

The YAP inhibitor verteporfin (Sigma, cat. no. SML0534) was injected into pregnant female mice at E15.5 (i.p., 100 μg/g body weight, 10 µl/g body weight). 22 h later, BrdU was injected (i.p., 1.5 mg). 2 h later, i.e. 24 h after verteporfin application, embryos were harvested. As a control, pregnant female mice at E15.5 were injected with vehicle, i.e. 10% DMSO in PBS (10 µl/g body weight).

### Basal cell carcinoma mouse model

Mice at the age of 7.5 weeks were injected with tamoxifen (1 mg; i.p.) for 5 consecutive days. The analyzed genotypes were “Gαs KO^ind^” (genotype K14-CreERT;*gnas*^flox/flox^;*plxnb1*^+/flox^;*plxnb2*^+/flox^) and “Gαs KO^ind^;PlexDKO^ind^” (genotype K14-CreERT;*gnas*^flox/flox^;*plxnb1*^flox/flox^;*plxnb2*^flox/flox^); “Patched1 KO^ind^” (genotype K14-CreERT;*ptch1*^flox/flox^;*plxnb1*^+/flox^;*plxnb2*^+/flox^) and “Patched1 KO^ind^;PlexDKO^ind^” (genotype K14-CreERT;*ptch1*^flox/flox^;*plxnb1*^flox/flox^;*plxnb2*^flox/flox^). Mice were regularly monitored for the appearance of skin lesions. The total observation period (from the start of tamoxifen injections to analysis) was 30 days. Both male and female mice were examined.

### Analysis of mitotic spindle angle

Paraffin-embedded sections of the murine epidermis at E16.5 were immunostained for phospho-histone H3 and stained with DAPI, and confocal images were acquired. The mitotic spindle angle, defined as the angle between the plane of the basal cell layer and the plane of mitosis (axis transecting two daughter nuclei) was quantified using ImageJ by an observer blinded to mouse genotypes.

### Quantitative image analysis

Segmentation analyses were done based on E-cadherin immunofluorescence stainings using the Tissue Analyzer plug-in in ImageJ. Cell area and shape (major axis/minor axis; roundness: 4×[area]/(*π* × [major axis]^2^) were quantified by using the “area” and “shape description” functions of ImageJ. The number of cells per area (cell density) was quantified manually.

To quantify mean fluorescence intensities, the segmented line tool of Fiji (line width 5 pixels) was used.

All quantifications were done by an observer blinded to genotypes.

For crowding experiments, images were analyzed using Fiji^93^. To quantify nuclear YAP, fields were randomly selected based exclusively on the presence of nuclei, as assessed by DAPI staining. Areas of interest were generated using automated thresholding of the DAPI staining, after which mean fluorescence intensities of nuclear YAP intensity was quantified within the areas of interest from maximum projection images obtained as described above. The measured mean nuclear intensities were normalized to DAPI intensity of the corresponding individual nuclei to account for possible unevenness in sample topology. To measure pMLC-2 intensity, pMLC-2 signal intensity at junctions was measured by automatic thresholding followed by subtraction of cytoplasmic background signal. Cell area was measured by manually tracing α18 fluorescence. Cell orientation was quantified by measuring the orientation angle (the angle between the long axis and a line parallel to the *x*-axis of the image which was aligned to the direction of stretch) of the major Feret diameter of areas of interest corresponding to traced α-18-positive adherens junctions.

### Micropatterns

Micropatterned adhesive surfaces were generated using the PRIMO optical module (Alvéole, France) controlled by the Leonardo plugin (V3.3, Alveole) mounted on a Nikon TI-E inverted microscope (Nikon Instruments) equipped with a Super Plan FLuor ×20 ELWD lens (Nikon) lens and a DMD-based UV (375 nm). To generate circular and square micropatterns, both 100 µm diameter, were projected onto plasma-cleaned (Corona Treater, ETP), PLLgPEG-passivated (0.1 mg/ml PLL-g-PEG (PLL (20)-g [3.5]- PEG (2), SuSoS) 35 mm glass-bottom dishes (Ibidi). Patterned areas were then washed multiple times with PBS and conjugated with a uniform coating of 10 µg/ml fibronectin and 38.75 µg/ml collagen for 1 h at +37 °C. The substrates were then washed with PBS, prior to seeding 100 K of mouse keratinocytes onto each 35 mm dish. Keratinocyte monolayers were allowed to proliferate on the patterns for 24 h, at which time they were subjected to AFM or fixed and processed for immunofluorescence and quantification analyses. For the analysis of YAP localization (Fig. 3d, e), only micropatterns with similar cell densities were compared.

### Crowding experiments

The custom-built uniaxial cell stretcher has been described in detail previously^42,94^. Polydimethylsiloxane (PDMS) elastomers were prepared from a two-component formulation (Sylgard 184, Dow Corning) by mixing base and crosslinker in a ratio of 40–1 (weight/weight) to obtain substrates with 50 kPa stiffness. Chambers were UV sterilized for 1 h and finally coated with fibronectin (20 µg/ml) in phosphate-buffered saline (PBS) for 1 h at 37 °C prior to cell seeding. 350,000 cells per elastomer (4 cm^2^) were seeded 16 h before experiment start. Before initiation of experiments, culture medium was replaced by medium containing 200 µM Ca^2+^ to promote cell–cell contact formation. Cells were then exposed to 20% static stretch for 12 h and fixed with 4% PFA either directly when under stretch or 5 or 15 min post release. For each independent experiment, cells from independent mice were used.

### Atomic force microscopy

AFM measurements were performed on micropatterns generated on glass bottom dishes (35 mm Ibidi). Measurements were done using a JPK NanoWizard 2 (Bruker Nano) atomic force microscope mounted on an Olympus IX73 inverted fluorescent microscope (Olympus) and operated via JPK SPM Control Software v.5. Spherical silicon dioxide beads with a diameter of 3.5 μm glued onto tipless silicon nitride cantilevers (NanoAndMore, CP-PNPL-SiO-B-5) with a nominal spring constant of 0.08 N m^−1^ were used. For all indentation experiments, forces of up to 4 nN were applied, and the velocities of cantilever approach and retraction were kept constant at 2 μm s^−1^ ensuring detection of elastic properties only. All analyses (>50 cells per experiment/condition) were performed with JPK Data Processing Software (Bruker Nano). Prior to fitting the Hertz model to obtain cell Young’s Modulus (Poisson’s ratio of 0.5), the offset was removed from the baseline, contact point was identified, and cantilever bending was subtracted from all force curves.

### Structured illumination microscopy (SIM)

Whole-mount immunofluorescence stainings of the epidermis of embryos at E15.5 were performed as described above. Widefield image z-stacks (500 nm slices) were acquired using structured illumination microscopy (SIM) (5xrotations) with a ZEISS ELYRA PS.1 setup (Laser lines: 561 nm/488 nm; sCMOS camera/ZEISS objective alpha Plan-Apochromat ×100/1.46 Oil DIC M27). SIM 3D reconstructions were processed using the implemented ZEN-Black software by ZEISS.

### Statistics and reproducibility

*p ≤ 0.05, **p ≤ 0.01, ***p ≤ 0.001, ****p ≤ 0.0001.

All experiments presented in the manuscript were repeated in at least three independent experiments/biological replicates.

### Reporting summary

Further information on research design is available in the Nature Research Reporting Summary linked to this article.

## Supplementary information

Supplementary Information

Reporting Summary

## Data Availability

The authors declare that the data supporting the findings of this study are available within the paper and its supplementary information files. Source data are provided with this paper.

## Source data

Source Data
