# Supplementary material for: Mechanochemical control of epidermal stem cell divisions by B-plexins
Source: Nat Commun. 2021 Feb 26;12:1308. doi: 10.1038/s41467-021-21513-9 (PMC7910479; doi:10.1038/s41467-021-21513-9)
Supplement: Supplementary file 1 — Supplementary Information [file 41467_2021_21513_MOESM1_ESM.pdf]

Nature Communications

Supplementary Information

Mechanochemical control of epidermal stem cell divisions by B-plexins

Chen Jiang, Ahsan Javed, Laura Kaiser, Michele M. Nava, Rui Xu, Dominique T. Brandt, Dandan Zhao, Benjamin Mayer, Javier Fernández-Baldovinos, Luping Zhou, Carsten Höß, Kovilen Sawmynaden, Arkadiusz Oleksy, David Matthews, Lee S. Weinstein, Heidi Hahn, Hermann-Josef Gröne, Peter Graumann, Carien M. Niessen, Stefan Offermanns, Sara A. Wickström, Thomas Worzfeld

Supplementary Figures 1-9
Supplementary Tables 1-2

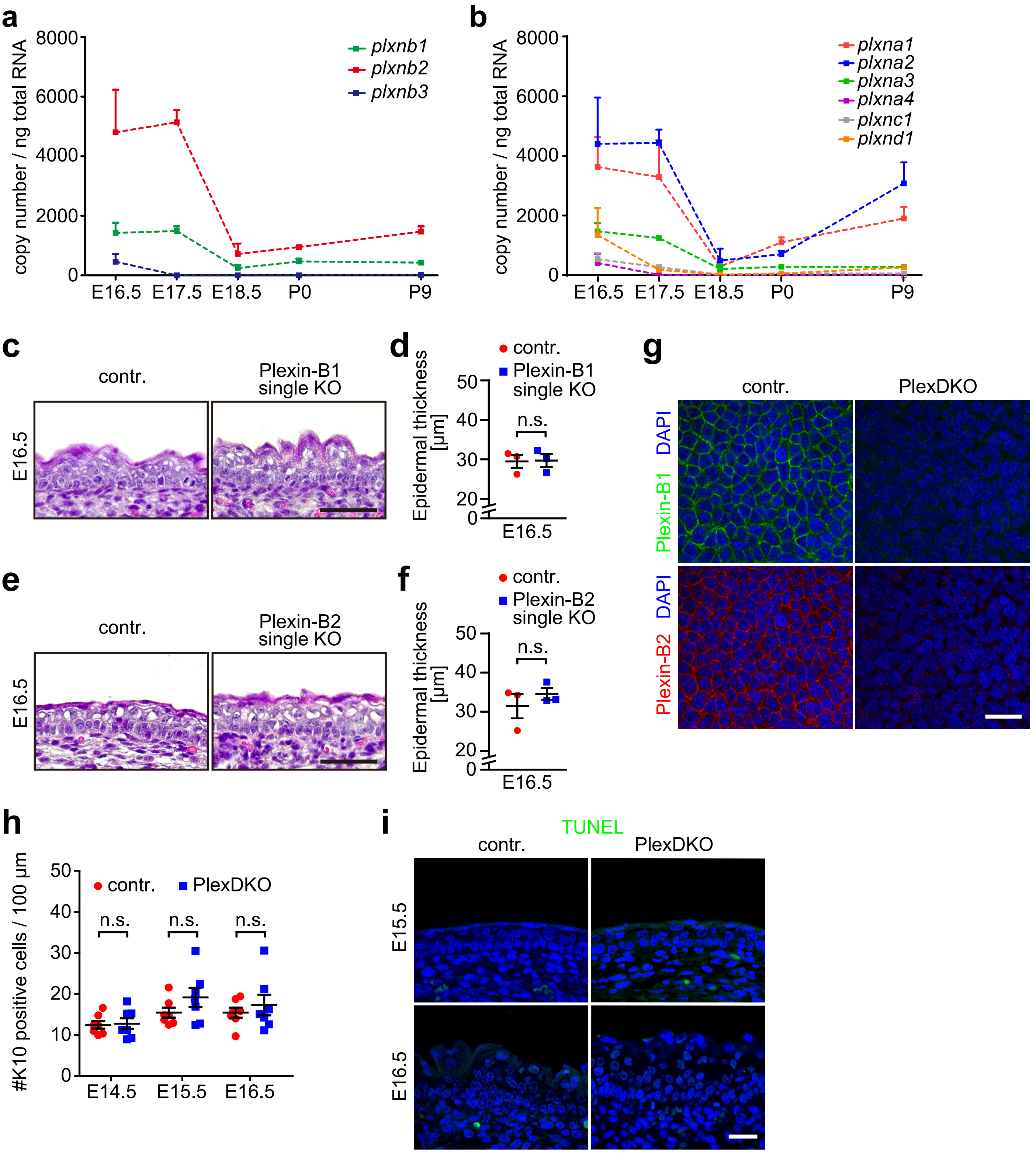

Supplementary Figure 1: a,**b,** mRNA expression levels in the murine epidermis of genes encoding plexins (*plxn*) at different time points of embryonic (E) and postnatal (P) development as determined by quantitative RT-PCR (mean ± s.e.m.; n=3 mice per time point). **c**, H&E stained histological sections of the epidermis of control ("contr."; genotype *plxnb1*$^{flox/flox}$) and Plexin-B1 single-deficient embryos ("Plexin-B1 single KO"; genotype K14-Cre;*plxnb1*$^{flox/flox}$) at E16.5. Scale bar, 25 µm. **d**, Quantification of the data in (c) (mean ± s.e.m.; n=3 embryos per genotype; p=0.9246; two-sided unpaired t-test). **e**, H&E stained histological sections of the epidermis of control ("contr."; genotype *plxnb2*$^{flox/flox}$) and Plexin-B2 single-deficient embryos ("Plexin-B2 single KO"; genotype K14-Cre;*plxnb2*$^{flox/flox}$) at E16.5. Scale bar, 25 µm. **f**, Quantification of the data in (e) (mean ± s.e.m.; n=3 embryos per genotype; p=0.4155; two-sided unpaired t-test). **g**, Confocal images of whole-mount immunostainings (top view) of murine epidermis at embryonic day 15.5 (E15.5) using anti-Plexin-B1 (green) and anti-Plexin-B2 antibodies (red). Scale bar, 25 µm. **h**, Quantification of K10-positive cells at the indicated embryonic time points (mean ± s.e.m.; n=7 mice per genotype and timepoint; p=0.860 for E14.5, p=0.187 for E15.5, p=0.520 for E16.5; two-sided unpaired t-test) **i**, TUNEL stainings of murine epidermis at E15.5 and E16.5. Blue: DAPI. Scale bar, 25 µm.

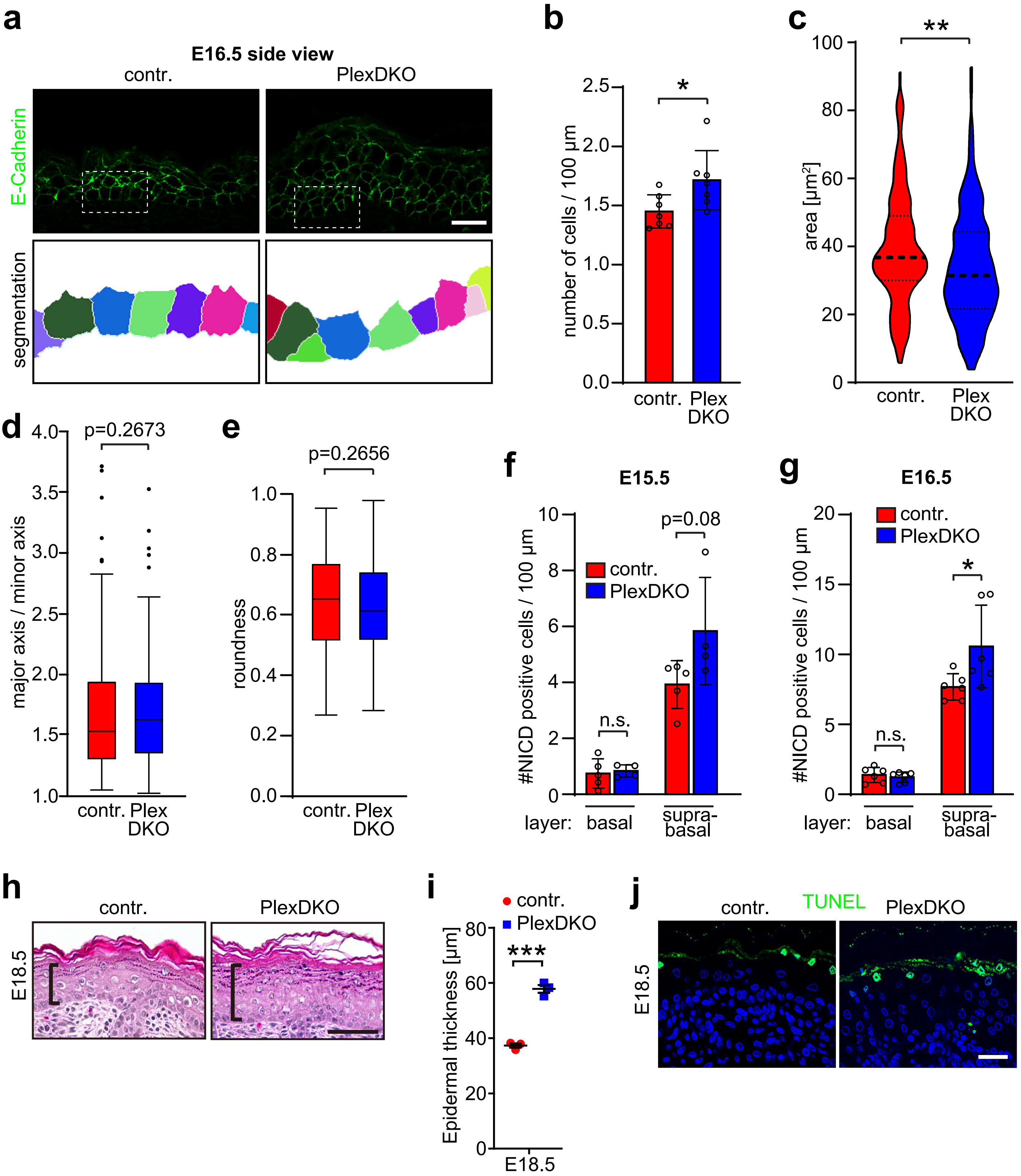

Supplementary Figure 2: **a**, Upper row: Confocal images of immunostainings (side view) of murine epidermis at E16.5 using an anti-E-cadherin antibody (green). Lower row: Segmentation analyses of the images depicted in the upper row. Scale bar, 25 µm. **b**, Quantification of the number of epidermal stem cells (basal layer) per distance (mean ± s.d.; n=7 mice per genotype; p=0.0309; two-sided unpaired t-test). **c**, Analysis of epidermal stem cell areas (violin plot with first quartile, median, and third quartile; control: n=166 from 7 mice, PlexDKO: n=178 from 7 mice; two-sided Mann-Whitney U test: p=0.0014). **d**,**e**, Analysis of cell shape anisotropy (box plot with minimum, first quartile, median, third quartile and maximum; control: n=166 from 7 mice, PlexDKO: n=178 from 7 mice; two-sided Mann-Whitney U test). **f**,**g**, Quantification of NICD-positive cells in the basal layer and in the suprabasal layers of the murine embryonic epidermis at (f) E15.5 and (g) E16.5 (mean ± s.d.; E15.5 control: n=5 mice, E15.5 PlexDKO: n=4 mice, E16.5: n=6 mice per genotype; p=0.08 for E15.5 suprabasal, p=0.045 for E16.5 suprabasal; two-sided unpaired t-test). **h**, H&E stained histological sections of murine epidermis at E18.5. Brackets indicate epidermal thickness. Scale bar, 50 µm. **i**, Quantification of the data in (h) (mean ± s.e.m.; n=3 mice per genotype; p=0.0002; two-sided unpaired t-test). **j**, TUNEL stainings of murine epidermis at E18.5. Blue: DAPI. Scale bar, 25 µm.

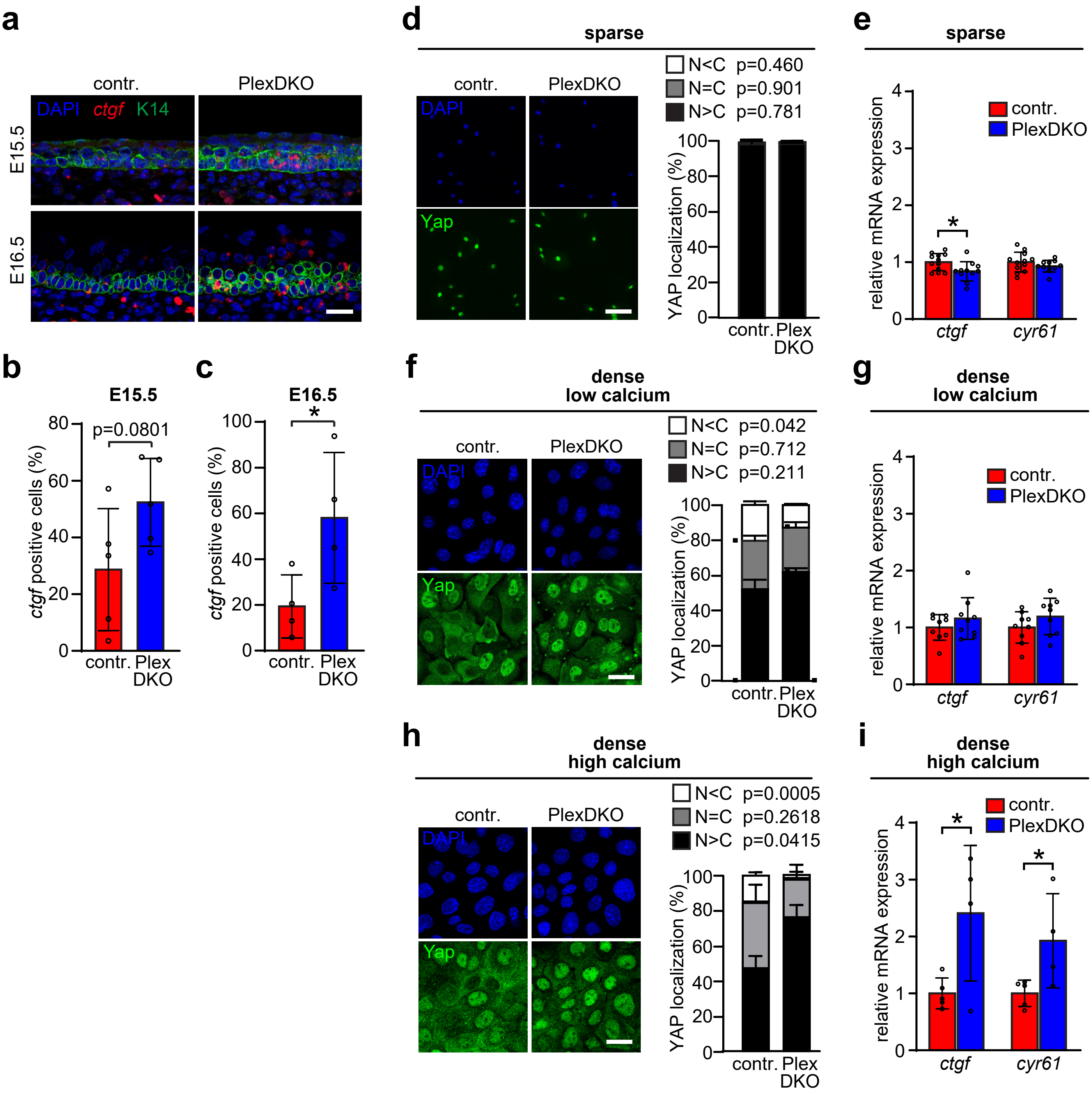

Supplementary Fig. 3: a, Confocal images of fluorescent in situ hybridizations for the YAP target gene *ctgf* (red) combined with an immunostaining for K14 (green). Scale bar, 25 µm. **b**,**c**, Quantification of the data in (a) (mean ± s.d.; E15.5: n=5 mice per genotype, p=0.0801; E16.5: n=4 mice per genotype, p=0.04939; two-sided unpaired t-test). **d**-**i**, Primary mouse keratinocytes cultured at different densities and calcium concentrations were immunostained for YAP (green). The left panels show representative images and quantifications of Yap localization. N: nuclear, C: cytoplasmic. Blue: DAPI. The right panels depict mRNA expression levels of the YAP target genes *ctgf* and *cyr61* as determined by quantitative RT-PCR. **d**,**e**, Primary mouse keratinocytes cultured at low density ("sparse") and 70 µM Ca^{2+}. Quantification of YAP localization: mean ± s.d.; control: n=859 from 4 mice, PlexDKO: n=1075 from 4 mice; two-sided unpaired t-test. Analysis of mRNA expression levels of YAP target genes: mean ± s.d.; control: n=12 mice, PlexDKO: n=11 mice; p=0.025 for *ctgf*, p=0.252 for *cyr61*; two-sided unpaired t-test. Scale bar, 100 µm. **f**,**g**, Primary mouse keratinocytes cultured at high density and 70 µM Ca^{2+}. Quantification of YAP localization: mean ± s.d.; control: n=468 from 4 mice, PlexDKO: n=309 from 3 mice; two-sided unpaired t-test. Analysis of mRNA expression levels of YAP target genes: mean ± s.d.; control: n=9 mice, PlexDKO: n=9 mice; p=0.283 for *ctgf*, p=0.192 for *cyr61*; two-sided unpaired t-test. Scale bar, 25 µm. **h**,**i**, Primary mouse keratinocytes cultured at high density and after 3 h of 1.8 mM Ca^{2+}. Quantification of YAP localization: mean ± s.d.; control: n=923 from 3 mice, PlexDKO: n=887 from 3 mice; two-sided unpaired t-test. Analysis of mRNA expression levels of YAP target genes: mean ± s.d.; control: n=5 mice, PlexDKO: n=4 mice; p=0.035 for *ctgf*, p=0.046 for *cyr61*; two-sided unpaired t-test. Scale bar, 25 µm.

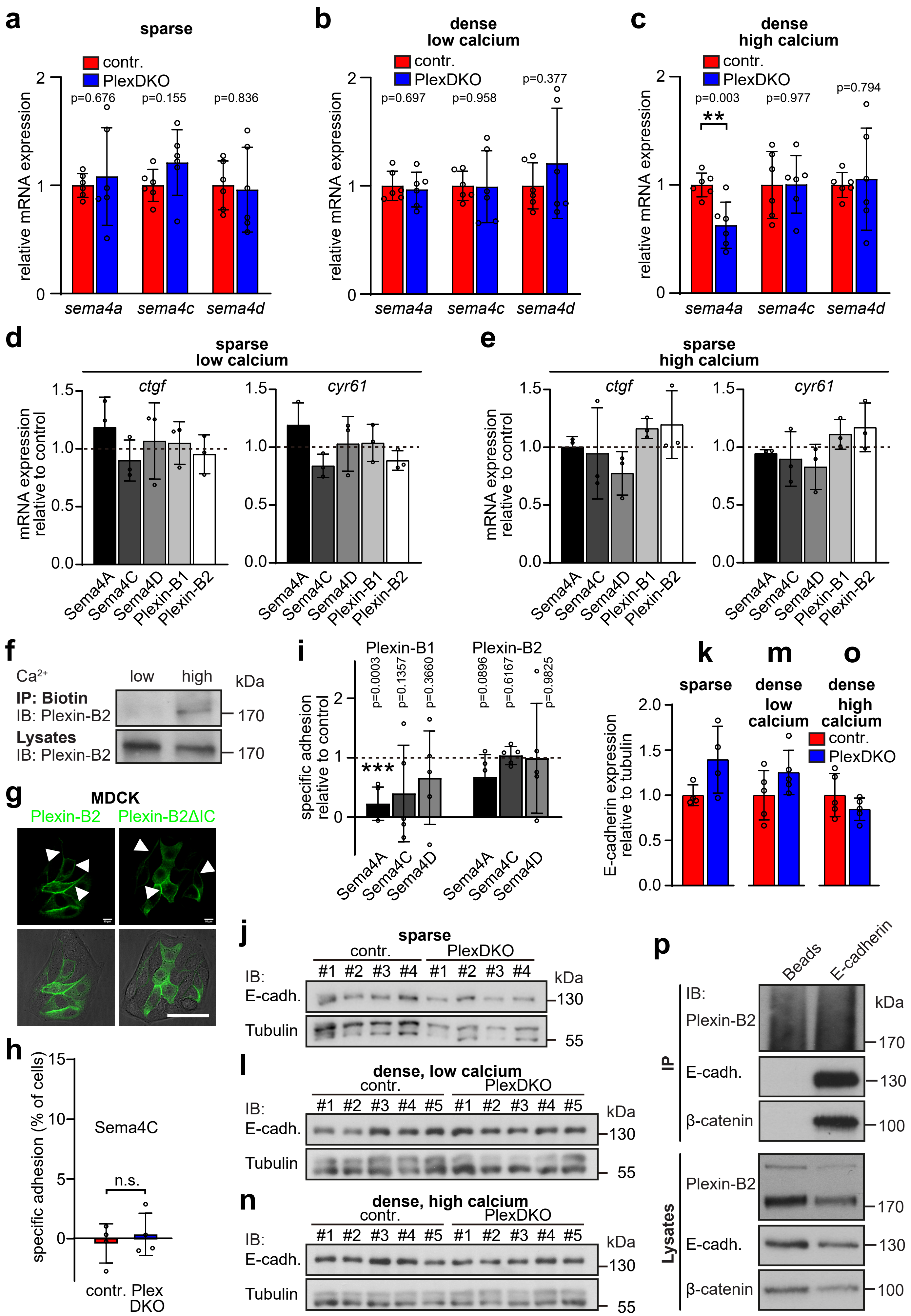

a
sparse
contr.
PlexDKO
p=0.676
p=0.155
p=0.836
relative mRNA expression
sema4a
sema4c
sema4d
b
dense
low calcium
p=0.697
p=0.958
p=0.377
c
dense
high calcium
p=0.003
**
p=0.977
p=0.794
d
sparse
low calcium
ctgf
cyr61
mRNA expression relative to control
Sema4A
Sema4C
Sema4D
Plexin-B1
Plexin-B2
e
sparse
high calcium
f
Ca2+
low
high
kDa
IP: Biotin
IB: Plexin-B2
Lysates
IB: Plexin-B2
170
g
MDCK
Plexin-B2
Plexin-B2ΔIC
h
Sema4C
n.s.
specific adhesion (% of cells)
contr.
Plex DKO
i
Plexin-B1
Plexin-B2
p=0.0003
p=0.1357
p=0.3660
p=0.0896
p=0.6167
p=0.9825
***
specific adhesion relative to control
k
m
o
sparse
dense low calcium
dense high calcium
E-cadherin expression relative to tubulin
j
sparse
contr.
PlexDKO
#1 #2 #3 #4
IB:
E-cadh.
Tubulin
130
55
l
dense, low calcium
#1 #2 #3 #4 #5
n
dense, high calcium
p
Beads
E-cadherin
IP
Lysates
Plexin-B2
E-cadh.
β-catenin
170
130
100

Supplementary Figure 4: a-c, Primary mouse keratinocytes of control or PlexDKO mice were cultured at different densities and calcium concentrations. Shown are relative mRNA expression levels of *sema4a*, *sema4c* and *sema4d* as determined by quantitative RT-PCR (mean ± s.d.; control: n=6 mice, PlexDKO: n=6 mice; two-sided unpaired t-test). **d**,**e**, Primary mouse keratinocytes cultured at sparse density and at 70 µM Ca^{2+} ("low calcium") or 1.8 mM Ca^{2+} ("high calcium") were incubated without or with recombinant Sema4A, Sema4C, Sema4D, Plexin-B1 or Plexin-B2 (25 nM). After 8 hours, the mRNA expression level of *ctgf* and *cyr61* relative to control (i.e. non-treated cells) was determined by quantitative RT-PCR (mean ± s.d.; *n*=3 independent mice). **f**, Primary mouse keratinocytes were cultured at low (70 µM) or overnight at high (1.8 mM) Ca^{2+} concentrations. Surface proteins were then biotinylated, precipitated using streptavidin agarose, and surface Plexin-B2 was visualized by Western blotting using an anti-Plexin-B2 antibody. **g**, MDCK renal tubular epithelial cells were engineered to stably express wildtype Plexin-B2 fused to GFP ("Plexin-B2") or mutant Plexin-B2 lacking the intracellular domain ("Plexin-B2ΔIC"). Shown are representative confocal images. Arrowheads point to cell-cell contacts. Scale bars, 10 µm. **h**, 96-well nitrocellulose plates were coated with the recombinant extracellular portion of murine Sema4C, and primary mouse keratinocytes of control or PlexDKO mice were allowed to adhere for 30 min at 37°C. Specific adhesion was quantified as described in Methods (*n*=4 per genotype; mean ± s.d.; p=0.555; two-sided unpaired t-test). **i**, 96-well nitrocellulose plates were coated with recombinant extracellular portions of Plexin-B1 or Plexin-B2, and primary mouse keratinocytes of control or PlexDKO mice were allowed to adhere for 30 min at 37°C in the absence (PBS; "control") or presence of Sema4A, Sema4C, Sema4D (25 nM). Specific adhesion was quantified as described in Methods, and is shown relative to control (PBS) (mean ± s.d.; *n*=5 independent mice; two-sided unpaired t-test). **j-o**, Primary mouse keratinocytes cultured at different densities and calcium concentrations were lysed, and E-cadherin ("E-cadh.") and α-tubulin ("Tubulin") were detected using respective antibodies. Each lane represents cells from a different mouse. Quantifications of the blots in (j), (l), (n) are shown in (k), (m), (o), respectively (mean ± s.d.; (k) p=0.088, (m) p=0.1646, (o) p=0.23; two-sided unpaired t-test). **p**, Primary mouse keratinocytes were cultured at high density and 1.8 mM Ca^{2+}. Cells were lysed, E-cadherin was immunoprecipitated (IP), and protein complexes were visualized by Western blotting (IB).

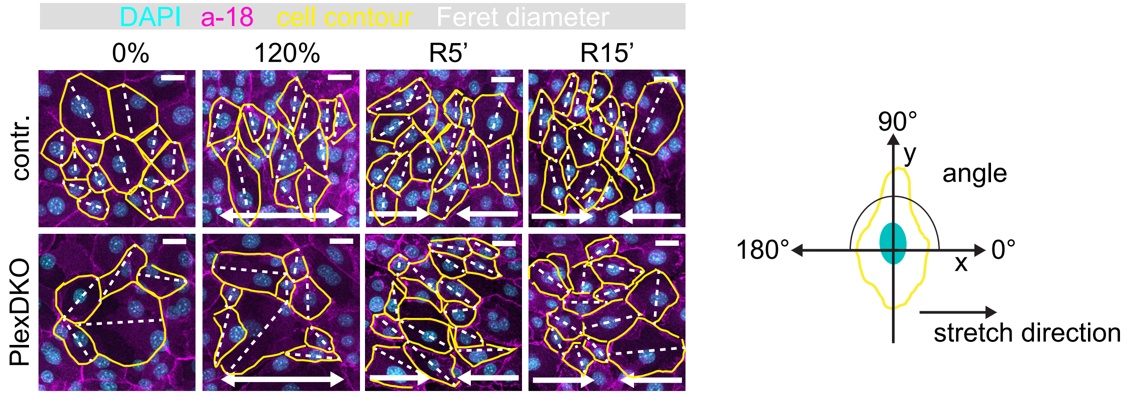

Supplementary Figure 5: Left: Shown are identical images as in Figure 4I with added cell contours as manually traced on α-catenin (α-18)-positive adherens junctions and the major Feret diameter. Right: Shown is a schematic illustrating the quantification of orientation angles. Scale bar, 20 µm.

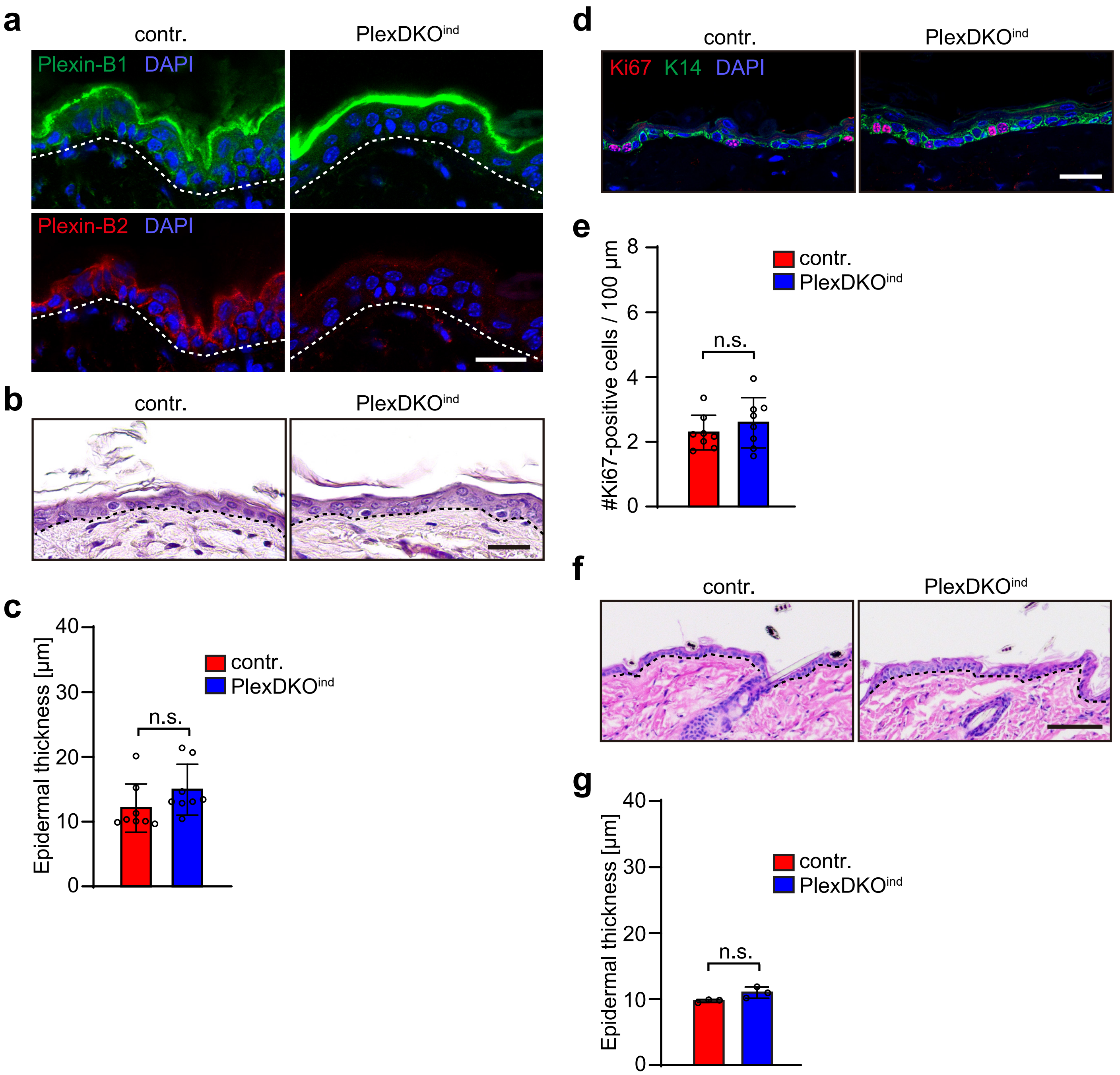

Supplementary Figure 6: a, Adult epidermis-specific tamoxifen-inducible Plexin-B1/Plexin-B2 double-knockout mice ("PlexDKO^{ind}"; genotype K14-CreERT;*plxnb1*$^{flox/flox}$;*plxnb2*$^{flox/flox}$) and respective control mice ("contr."; genotype *plxnb1*$^{flox/flox}$;*plxnb2*$^{flox/flox}$) were treated with tamoxifen for 5 consecutive days, and the epidermis was analyzed. Shown are confocal images of immunostainings using anti-Plexin-B1 (green) or anti-Plexin-B2 (red) antibodies. Scale bar, 25 µm. **b**, H&E stained histological sections of adult skin of mice with the indicated genotypes 4 weeks after tamoxifen treatment. Dashed lines indicate the basement membrane. Scale bar, 25 µm. **c**, Quantification of epidermal thickness (mean ± s.d.; n=8 mice per genotype; p=0.159; two-sided unpaired t-test). **d**, Confocal images of immunostainings of the skin using anti-Ki67 (red) and anti-K14 antibodies (green). Scale bar, 25 µm. **e**, Quantification of the data in (d) (mean ± s.d.; n=8 mice per genotype; p=0.3821; two-sided unpaired t-test). **f**, H&E stained histological sections of adult skin of mice with the indicated genotypes 17 weeks after tamoxifen treatment. Dashed lines indicate the basement membrane. Scale bar, 100 µm. **g**, Quantification of epidermal thickness (mean ± s.d.; n=3 mice per genotype; p=0.068; two-sided unpaired t-test).

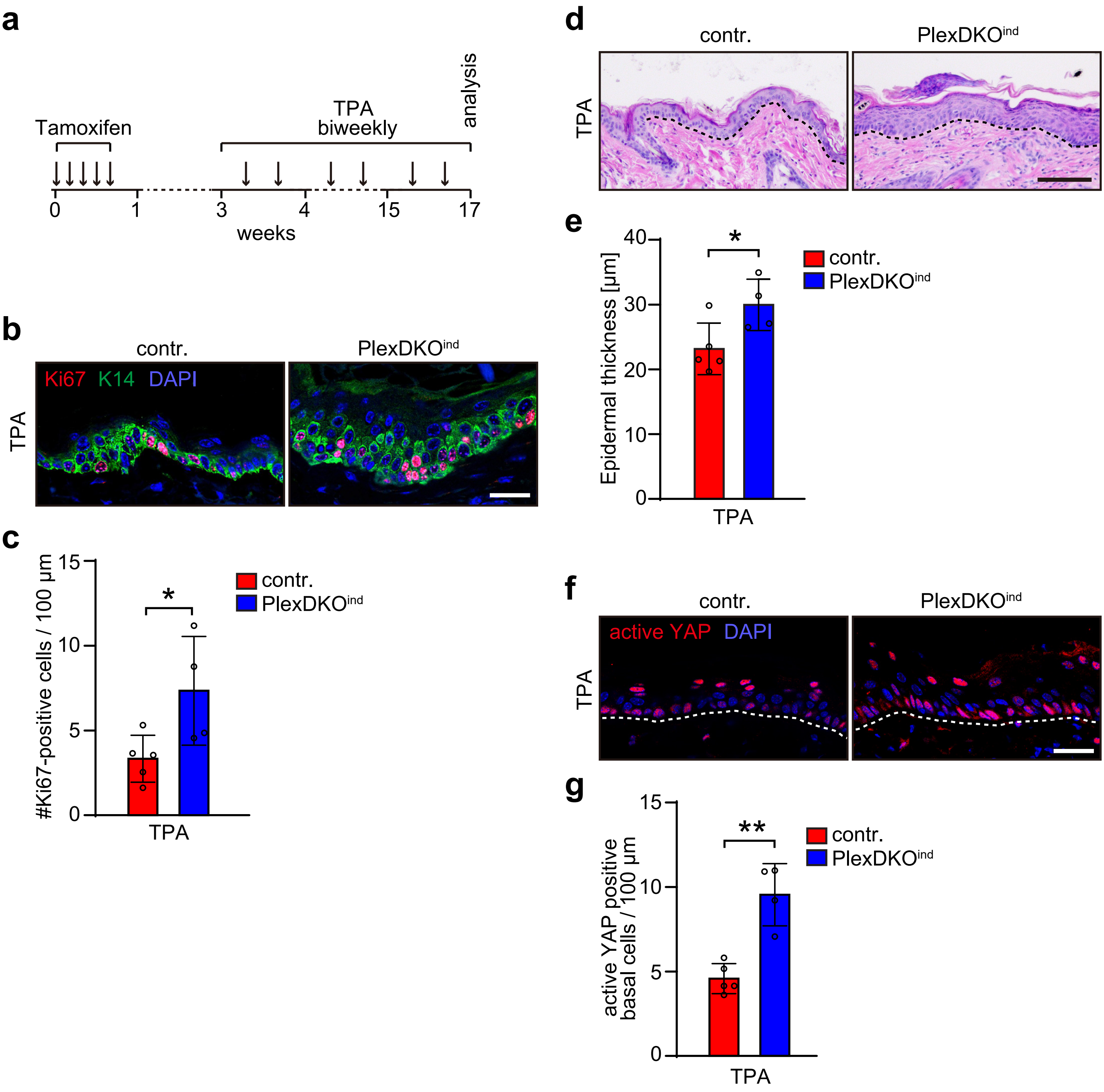

Supplementary Figure 7: a, Timeline illustrating the treatment regimen of mice with tamoxifen and 12-O-Tetradecanoyl-phorbol-13-acetate (TPA). **b-g**, Mice with the indicated genotypes were treated according to the scheme depicted in (a). **b**, Confocal images of immunostainings of the skin using anti-Ki67 (red) and anti-K14 antibodies (green). Scale bar, 25 µm. **c**, Quantification of the data in (b) (mean ± s.d.; contr.: n=5 mice, PlexDKO: n=4 mice; p=0.0381; two-sided unpaired t-test). **d**, H&E stained histological sections of the skin. Scale bar, 100 µm. **e**, Quantification of epidermal thickness (mean ± s.d.; contr.: n=5 mice, PlexDKO: n=4 mice; p=0.0379; two-sided unpaired t-test). **f**, Confocal images of immunostainings of the skin using an anti-active YAP (red) antibody. Scale bar, 25 µm. **g**, Quantification of the data in (f) (mean ± s.d.; contr.: n=5 mice, PlexDKO: n=4 mice; p=0.0011; two-sided unpaired t-test).

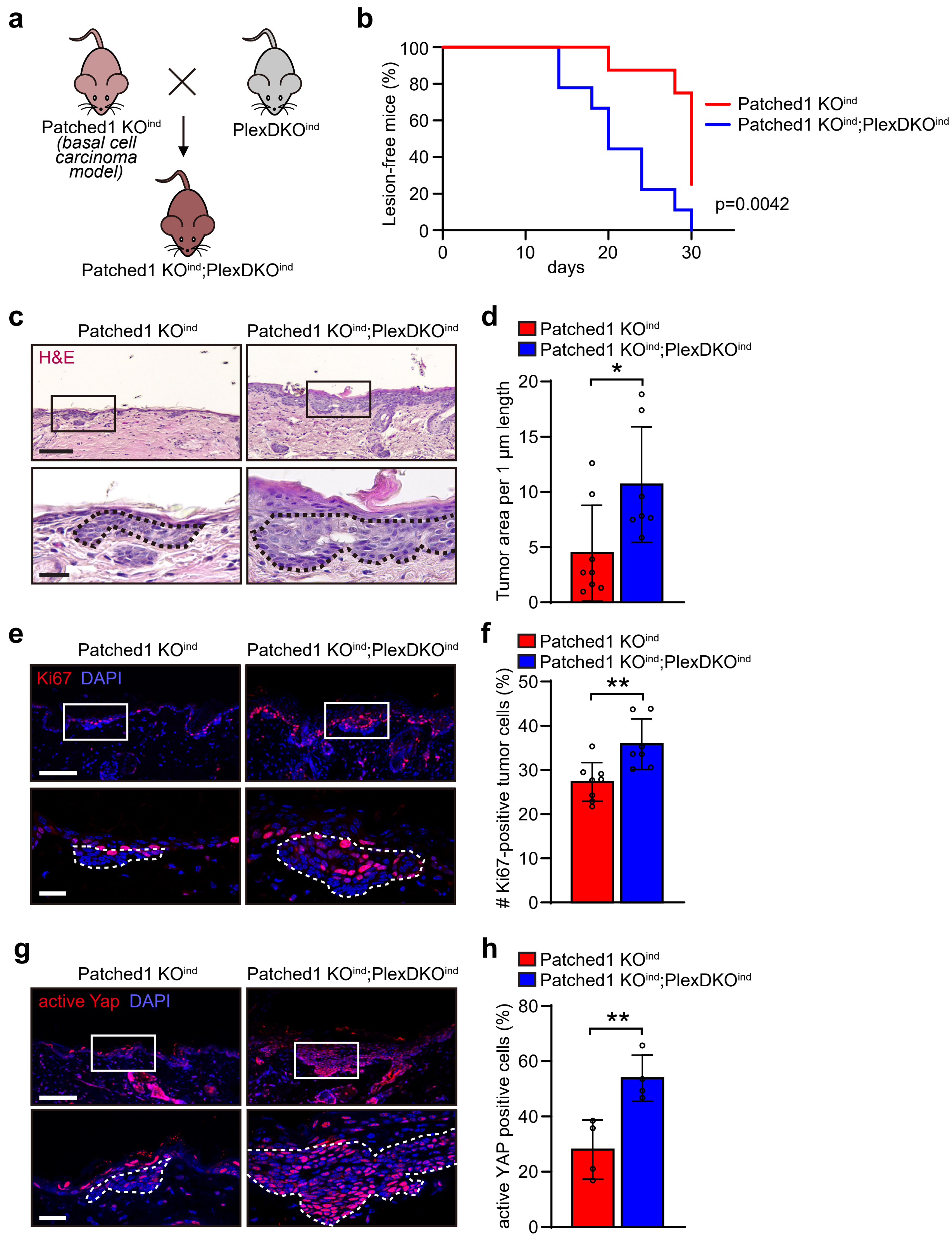

Supplementary Figure 8: **a**, Schematic illustration of the generation of epidermis-specific tamoxifen-inducible Patched-1/Plexin-B1/Plexin-B2 triple-deficient mice. **b**, Kaplan-Meier curves representing the percentage of lesion-free mice. Time point "0" indicates the start of tamoxifen treatment (Patched1 KO^{ind} mice: n=8, Patched1 KO^{ind};PlexDKO^{ind} mice: n=8; Mantel-Cox test for Patched1 KO^{ind} compared to Patched1 KO^{ind};PlexDKO^{ind}). **c**-**h**, Histological/immunofluorescence stainings (left panels) and respective quantifications (right panels) of adult murine skin of mice with the indicated genotypes 30 days after treatment with tamoxifen. Boxed areas are magnified in the lower rows. Tumors are marked by dashed lines. **c**, H&E stain of histological sections. Scale bar, 100 µm (upper row), 25 µm (lower row). **d**, Quantification of tumor areas (mean ± s.d.; Patched1 KO^{ind} mice: n=8, Patched1 KO^{ind};PlexDKO^{ind} mice: n=7; p=0.0255; two-sided unpaired t-test). **e**, Confocal images of immunostainings using an anti-Ki67 antibody (red). Scale bar, 100 µm (upper row), 25 µm (lower row). **f**, Quantification of Ki67-positive cells (mean ± s.d.; Patched1 KO^{ind} mice: n=8, Patched1 KO^{ind};PlexDKO^{ind} mice: n=7; p=0.0059; two-sided unpaired t-test). **g**, Confocal images of immunostainings using an anti-active YAP antibody (red). Scale bar, 100 µm (upper row), 25 µm (lower row). **h**, Quantification of cells positive for active YAP (mean ± s.d.; Patched1 KO^{ind} mice: n=4, Patched1 KO^{ind};PlexDKO^{ind} mice: n=4; p=0.0089; two-sided unpaired t-test).

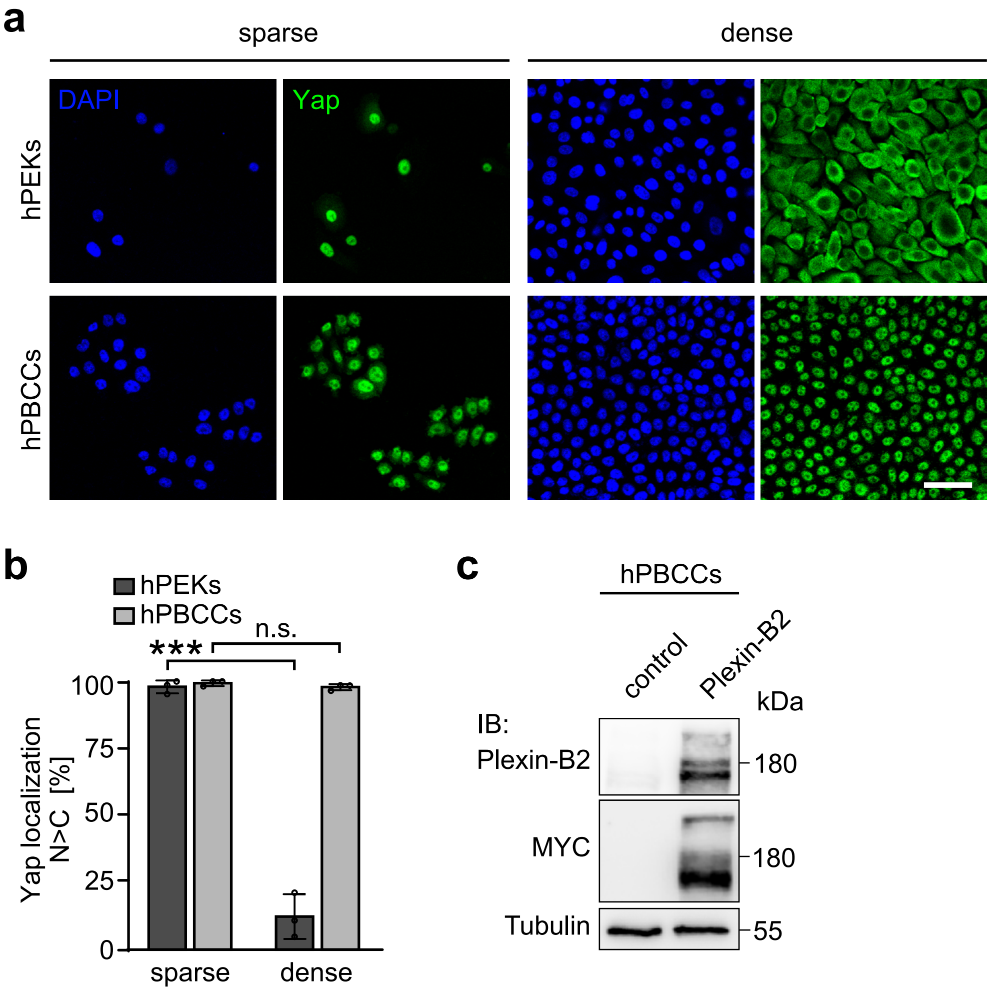

Supplementary Figure 9: a,**b**, hPEKS and hPBCCs were cultured at different densities and immunostained for YAP (green). Shown are representative confocal images in (a) and a quantification of YAP localization in (b) (mean ± s.d.; hPEKs sparse: *n*=269 cells, hPEKs dense: *n*=947 cells, hPBCCs sparse: *n*=452 cells, hPBCCs dense: *n*=1909 cells, from 3 independent experiments; p<0.0001 for hPEKs, p=0.1724 for hPBCCs; two-sided unpaired t-test). N: nuclear, C: cytoplasmic. Blue: DAPI. Scale bar, 50 µm. **c**, Human primary basal cell carcinoma cells (hPB-CCs) expressing either GFP ("control") or triple-myc-tagged mouse Plexin-B2 ("Plexin-B2) were lysed, and proteins were detected using the indicated antibodies.

Supplementary Table 1: Primers used for absolute quantification of gene expression

species	gene	primer	sequence
mouse	*plxna1*	mPA1_forward	5’-GGGATGCTGCAGGTGTATTC-3’
		mPA1_reverse	5’-CACCACCGATACCCACAAT-3’
	plxna2	mPA2_forward	5’-GGACTATGAGCTCCACAGTGATT-3’
		mPA2_reverse	5’-AGGGTGTCAGAGGGAATCTTG-3’
	plxna3:	mPA3_forward	5’-GTTACAGCTGCTGGTCATGC-3’
		mPA3_reverse	5’-GCACCCTCCTATGGTGAAGA-3’
	plxna4	mPA4_forward	5’-CAGCATGCAGGCTTTGTG-3’
		mPA4_reverse	5’-AAGAGCGGGCCATGAACT-3’
	plxnb1	mPB1_forward	5’-CTGCCAGGGTGGTCGTTA-3’
		mPB1_reverse	5’-ACCTCCTTGGACGTGGCTA-3’
	plxnb2	mPB2_forward	5’-CAAACACCAGGTGGAAAAGG-3’
		mPB2_reverse	5’- GCCTGTGTCATTGAGGGTGT-3’
	plxnb3	mPB3_forward	5’-GCCAGGCCTTTAATGATGTG-3’
		mPB3_reverse	5’-AGAAGGGTCAGGGTTGCAG-3’
	plxnc1	mPC1_forward	5’-GCCAGATTCAGAAGCATTCC-3’
		mPC1_reverse	5’-TGAACTT-GTGTTTCCCTCGAT-3’
	plxnd1	mPD1_forward	5’-CGGTTCCGCCTAGACTACCT-3’
		mPD1_reverse	5’-TGGGTGATGTTTGATCCACTT-3’

Supplementary Table 2: Primers used for relative quantification of gene expression

species	gene	primer	sequence
mouse	*ctgf*	mCTGF_forward	5'-TGACCTGGAGGAAAACATTAAGA-3'
		mCTGF_reverse	5'-AGCCCTGTATGTCTTCACACTG-3'
human	*ctgf*	hCTGF_forward	5'-AGGAGTGGGTGTGTGACGA-3'
		hCTGF_reverse	5'-CCAGGCAGTTGGCTCTAATC-3'
mouse	*cyr61*	mCYR61_forward	5'-GGATCTGTGAAGTGCGTCCT-3'
		mCYR61_reverse	5'-CTGCATTTCTTGCCCTTTTT-3'
human	*cyr61*	hCYR61_forward	5'-AAACCCGGATTTGTGAGGT-3'
		hCYR61_reverse	5'-GCTGCATTTCTTGCCCTTT-3'
mouse	*sema4a*	mSEMA4A_forward	5'-GGACCACCTTCCTCAAAGC-3'
		mSEMA4A_reverse	5'-GTGGCGGATGATGTTGAAT-3'
mouse	*sema4c*	mSEMA4C_forward	5'-TCTTTATGACACGGGACTCCA-3'
		mSEMA4C_reverse	5'-CGATAGGGTCCAGAGTGACG-3'
mouse	*sema4d*	mSEMA4D_forward	5'-GGCACTCTGCATGTACTTTCC-3'
		mSEMA4D_reverse	5'-TGTGCGCCTACACACTGG-3'
mouse	*gapdh*	mGAPDH_forward	5'-AGGTCGGTGTGAACGGATTTG-3'
		mGAPDH_reverse	5'-TGTAGACCATGTAGTTGAGGTCA-3'
human	*actb* (beta-actin)	hACTB_forward	5'-CCAGAGGCGTACAGGGATAG-3'
		hACTB_reverse	5'-CCAACCGCGAGAAGATGA-3'
