# Supplementary material for: Mechanochemical control of epidermal stem cell divisions by B-plexins
Source: Nat Commun. 2021 Feb 26;12:1308. doi: 10.1038/s41467-021-21513-9 (PMC7910479; doi:10.1038/s41467-021-21513-9)
Supplement: Supplementary file 2 — Reporting Summary [file 41467_2021_21513_MOESM2_ESM.pdf]

nature research

Corresponding author(s): Thomas Worzfeld

Last updated by author(s): Jan 12, 2021

Reporting Summary

Nature Research wishes to improve the reproducibility of the work that we publish. This form provides structure for consistency and transparency in reporting. For further information on Nature Research policies, see our Editorial Policies and the Editorial Policy Checklist.

Statistics

For all statistical analyses, confirm that the following items are present in the figure legend, table legend, main text, or Methods section.

n/a	Confirmed	
☐	☒	The exact sample size (*n*) for each experimental group/condition, given as a discrete number and unit of measurement
☐	☒	A statement on whether measurements were taken from distinct samples or whether the same sample was measured repeatedly
☐	☒	The statistical test(s) used AND whether they are one- or two-sided<br>*Only common tests should be described solely by name; describe more complex techniques in the Methods section.*
☒	☐	A description of all covariates tested
☐	☒	A description of any assumptions or corrections, such as tests of normality and adjustment for multiple comparisons
☐	☒	A full description of the statistical parameters including central tendency (e.g. means) or other basic estimates (e.g. regression coefficient) AND variation (e.g. standard deviation) or associated estimates of uncertainty (e.g. confidence intervals)
☐	☒	For null hypothesis testing, the test statistic (e.g. *F*, *t*, *r*) with confidence intervals, effect sizes, degrees of freedom and *P* value noted<br>*Give P values as exact values whenever suitable.*
☒	☐	For Bayesian analysis, information on the choice of priors and Markov chain Monte Carlo settings
☒	☐	For hierarchical and complex designs, identification of the appropriate level for tests and full reporting of outcomes
☒	☐	Estimates of effect sizes (e.g. Cohen's *d*, Pearson's *r*), indicating how they were calculated

Our web collection on statistics for biologists contains articles on many of the points above.

Software and code

Policy information about availability of computer code

Data collection	Zeiss ZEN was used for confocal and SIM image acquisition. Leica LAS was used for the acquisition of histological and live cell images. BioRad Image Lab was used for immunoblot image acquisition. Quantitative PCR data were collected with the Bio-Rad CFX system.
Data analysis	GraphPad Prism 8.0 or Microsoft Excel were used to perform statistical analyses. Segmentation analyses were done based on E-cadherin immunofluorescence stainings using the Tissue Analyzer plug-in in ImageJ. Cell area and shape were quantified by using the “area” and “shape description” functions of ImageJ. The number of cells per area (cell density) was quantified manually. To quantify mean fluorescence intensities, the segmented line tool of ImageJ (line width 5 pixels) was used. Cell orientation and cell division orientation were quantified by measuring the orientation angle by ImageJ.

For manuscripts utilizing custom algorithms or software that are central to the research but not yet described in published literature, software must be made available to editors and reviewers. We strongly encourage code deposition in a community repository (e.g. GitHub). See the Nature Research guidelines for submitting code & software for further information.

Data

Policy information about availability of data

All manuscripts must include a data availability statement. This statement should provide the following information, where applicable:

- Accession codes, unique identifiers, or web links for publicly available datasets
- A list of figures that have associated raw data
- A description of any restrictions on data availability

The authors declare that the data supporting the findings of this study are available within the paper and its supplementary information files.

Field-specific reporting

Please select the one below that is the best fit for your research. If you are not sure, read the appropriate sections before making your selection.

[x] Life sciences [ ] Behavioural & social sciences [ ] Ecological, evolutionary & environmental sciences

For a reference copy of the document with all sections, see nature.com/documents/nr-reporting-summary-flat.pdf

Life sciences study design

All studies must disclose on these points even when the disclosure is negative.

Sample size	Sample sizes were chosen based on previous experience with similar experiments to test for statistical significance.
Data exclusions	No data were excluded.
Replication	All attempts of replication were successful.
Randomization	Samples were allocated based on their genotypes as described in the paper.
Blinding	The quantifications and scorings were done by an observer blinded to mouse genotypes.

Reporting for specific materials, systems and methods

We require information from authors about some types of materials, experimental systems and methods used in many studies. Here, indicate whether each material, system or method listed is relevant to your study. If you are not sure if a list item applies to your research, read the appropriate section before selecting a response.

Materials & experimental systems

n/a	Involved in the study	
[ ]	[x]	Antibodies
[ ]	[x]	Eukaryotic cell lines
[x]	[ ]	Palaeontology and archaeology
[ ]	[x]	Animals and other organisms
[x]	[ ]	Human research participants
[x]	[ ]	Clinical data
[x]	[ ]	Dual use research of concern

Methods

n/a	Involved in the study	
[x]	[ ]	ChIP-seq
[x]	[ ]	Flow cytometry
[x]	[ ]	MRI-based neuroimaging

Antibodies

Antibodies used

The following primary antibodies were used:

guinea pig polyclonal anti-keratin 14 (1:200, Progen, cat. no. GP-CK14),
rabbit polyclonal anti-keratin 14 (1:200, Biolegend, cat. no. #905301),
rabbit polyclonal anti-keratin 10 (1:200, Bio-legend, cat. no. #905401),
rabbit polyclonal anti-Ki67 (1:200, Abcam, cat. no. ab15580),
bioti-nylated monoclonal anti-BrdU (1:100, Biolegend, cat. #339810),
rabbit polyclonal anti-phospho-histone H3 (1:200, Cell Signaling, cat. no. #9701),
mouse monoclonal anti-Plexin-B1 (for Western Blot; 1:500, Santa Cruz, cat. no. sc-28372),
armenian hamster monoclonal anti-mouse Plexin-B2 (for immunostainings; 1:200, eBioscience, cat. #14-5665-85),
sheep poly-clonal anti-mouse Plexin-B2 (for Western Blot; 1:500, R&D Systems, cat. no. AF6836),
sheep polyclonal anti-human Plexin-B2 (for Western Blot; 1:500, R&D Systems, cat. no. AF5329),
rabbit monoclonal anti-E-cadherin (1:200, Cell Signaling, cat. no. #3195),
rabbit polyclonal anti--catenin (1:200, Invitrogen, cat. no. 71-1200),
anti-α-catenin a-18 (for immunostainings on elastomers; 1:10000, 74),
mouse monoclonal anti-β-catenin (1:1000; BD Bioscience, cat. no. 610153),
rabbit monoclonal anti-cleaved Notch1 (NICD, 1:100, Cell Signaling, cat. no. #4147),
rabbit monoclonal anti-Yap antibody (1:200, Cell Signaling, cat. no. #14074),
mouse monoclonal anti-Yap (for immunostainings on elastomers; 1:300, Santa Cruz; sc-101199),
rabbit monoclonal anti-active Yap (1:200, Abcam, cat. no. ab205270),
rabbit polyclonal anti-phospho-myosin light chain 2 (Ser19) (1:200, Cell Signaling, cat. no. #3671),
rabbit polyclonal anti-phospho-myosin light chain 2 (Thr18/Ser19) (for immunostainings on elastomers; 1:200, Cell Signaling; cat. no. #3674),

rabbit polyclonal anti-c-myc–Peroxidase (for Western Blot; 1:2000, Sigma, cat. no. A5598),
mouse monoclonal anti-myc (for immunostainings; 1:500, Cell Signaling, cat. no. #2276),
rabbit monoclonal anti-α-tubulin (1:1000, Cell Signaling, cat. no. #2125).
An anti-Plexin-B1 antibody was raised against the extracellular domain of Plexin-B1.

The following secondary antibodies were used:
Cy3-conjugated anti-armenian hamster (1:200; Jackson ImmunoResearch, cat. no. 127-165-160),
AlexaFluor 488-conjugated anti-guinea pig (1:200; Invitrogen, cat. no. A11073),
AlexaFluor 488-conjugated anti-rabbit (1:200; Invitrogen, cat. no. R37118),
AlexaFluor 555-conjugated anti-rabbit (1:200; Invitrogen, cat. no. A21429),
AlexaFluor 555-conjugated anti-mouse (1:200; Invitrogen, cat. no. A21424),
AlexaFluor 488-conjugated anti-mouse (1:200; Invitrogen, cat. no. A11029),
HRP-conjugated anti-sheep (1:5000, Invitrogen, cat. no. A16041).

Validation

The self-made anti-Plexin-B1 antibody was validated by using Plexin-B1 knockout mice (Figure S1g).
The anti-α-catenin a-18 was validated in a previous report: Nat Cell Biol 12, 533-542 (2010).
All commercial antibodies have been tested for specificity by their respective suppliers.

Eukaryotic cell lines

Policy information about cell lines

Cell line source(s)

Primary mouse keratinocytes were isolated as described in Methods.
Human primary epidermal keratinocytes (hPEKs) were purchased from CellnTec.
Human primary basal cell carcinoma cells (hPBCCs) were purchased from Celprogen.
MDCK cells and HEK293T were originally from ATCC.

Authentication

Primary mouse keratinocytes were verified by checking morphological features and the expression of Keratin 14. hPEKs and hPBCCs were verified by the respective suppliers. MDCK cells and HEK293T were checked by their morphological features, but have not been authenticated on a molecular level.

Mycoplasma contamination

All cell lines were tested negative for mycoplasma contamination.

Commonly misidentified lines
(See ICLAC register)

No commonly misidentified cell lines were used in this paper.

Animals and other organisms

Policy information about studies involving animals; ARRIVE guidelines recommended for reporting animal research

Laboratory animals

To generate mice lacking Plexin-B1 and Plexin-B2 specifically in the epidermis, mice carrying conditional alleles of Plexin-B1 and Plexin-B2 were crossed with mice expressing Cre constitutively under the control of the keratin 14 (K14) promoter, or with mice expressing CreERT under the control of the K14 promoter (Tg(KRT14-cre/ERT)20Efu/J, The Jackson Laboratory, Stock No. 005107). Mice carrying floxed alleles of Gαs or of Patched1 have been described previously. All mice used in this study were on a C57BL/6 genetic background. Mice were housed under a 12-h light-dark cycle with free access to food and water, and under specific pathogen-free conditions.

Wild animals

No wild animals were used.

Field-collected samples

No Field-collected samples were used.

Ethics oversight

All procedures were performed in accordance with German Animal Welfare legislation.

Note that full information on the approval of the study protocol must also be provided in the manuscript.
